# Thioguanosine Conversion Enables mRNA‐Lifetime Evaluation by RNA Sequencing Using Double Metabolic Labeling (TUC‐seq DUAL)

**DOI:** 10.1002/anie.201916272

**Published:** 2020-02-28

**Authors:** Catherina Gasser, Isabel Delazer, Eva Neuner, Katharina Pascher, Karl Brillet, Sarah Klotz, Lukas Trixl, Maximilian Himmelstoß, Eric Ennifar, Dietmar Rieder, Alexandra Lusser, Ronald Micura

**Affiliations:** ^1^ Institute of Organic Chemistry and Center for Molecular Biosciences Leopold-Franzens University Innrain 80 6020 Innsbruck Austria; ^2^ Institute of Molecular Biology Biocenter Medical University of Innsbruck Innrain 82 6020 Innsbruck Austria; ^3^ Université de Strasbourg Architecture et Réactivité de l'ARN—CNRS UPR 9002 Institut de Biologie Moléculaire et Cellulaire 67000 Strasbourg France; ^4^ Institute of Bioinformatics Biocenter Medical University of Innsbruck Innrain 82 6020 Innsbruck Austria

**Keywords:** gene expression, nucleoside modifications, oligonucleotides, RNA sequencing, RNA structures

## Abstract

Temporal information about cellular RNA populations is essential to understand the functional roles of RNA. We have developed the hydrazine/NH_4_Cl/OsO_4_‐based conversion of 6‐thioguanosine (6sG) into A′, where A′ constitutes a 6‐hydrazino purine derivative. A′ retains the Watson–Crick base‐pair mode and is efficiently decoded as adenosine in primer extension assays and in RNA sequencing. Because 6sG is applicable to metabolic labeling of freshly synthesized RNA and because the conversion chemistry is fully compatible with the conversion of the frequently used metabolic label 4‐thiouridine (4sU) into C, the combination of both modified nucleosides in dual‐labeling setups enables high accuracy measurements of RNA decay. This approach, termed TUC‐seq DUAL, uses the two modified nucleosides in subsequent pulses and their simultaneous detection, enabling mRNA‐lifetime evaluation with unprecedented precision.

## Graphical abstract

**Novel purine‐nucleoside conversion** chemistry paves the way for mRNA‐lifetime evaluation with unprecedented precision. The technique, termed TUC‐seq DUAL, uses two modified nucleosides, 6‐thioguanosine (6sG) and 4‐thiouridine (4sU), in subsequent pulses for metabolic labeling. Chemical treatment induces G‐to‐A and U‐to‐C mutations that are simultaneously analyzed upon RNA sequencing. Thus, synthesis and decay of mRNA can be clearly distinguished.

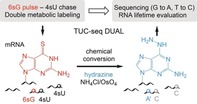

## Introduction

RNA maintains many important functions in the cell with most of the RNA‐involving processes being tightly regulated. Whole transcriptome sequencing (RNA‐sequencing) reveals the presence and quantities of RNAs at a given time.^1^, ^2^, ^3^, ^4^, ^5^, ^6^, ^7^, ^8^, ^9^, ^10^ To dissect variations in gene expression patterns that originate from changes in RNA transcription, processing, and decay, more advanced methods are needed. Thus far, the most widely applied approach to determine RNA dynamics involves metabolic labeling of RNA with 4‐thiouridine (4sU) in living cells.^11^, ^12^, ^13^, ^14^, ^15^ The strength of this strategy is that by targeting only newly synthesized 4sU‐containing transcripts, the time resolution to follow the biological process of interest increases significantly. In the original protocol, the isolated total RNA was thiol‐specifically biotinylated, followed by separation into newly transcribed and pre‐existing, unlabeled RNA.^16^ With TUC‐seq, we have recently published the first protocol that eliminates bothersome RNA enrichment steps by quantitatively converting 4‐thiouridine into native cytosine using oxidative OsO_4_‐based substitution chemistry, allowing for direct detection of newly synthesized transcripts containing U‐to‐C mutations by sequencing.^17^, ^18^ Two related methods, SLAM‐seq and TimeLapse‐seq,^19^, ^20^ have also been reported; however, both approaches modify 4sU to a non‐native pyrimidine analogue, an S‐alkylated 4sU thioiminoester and an *N*‐trifluoroethyl‐modified cytosine, respectively. These two pyrimidine derivatives give rise to apparent U‐to‐C mutations in RNA‐seq analysis although one has to keep in mind that downstream processing can be complicated or even impaired by the modification. All three 4sU‐based RNA‐seq methods have been validated independently by meaningful applications, including detection of endogenous 4sU in tRNAs,^17^, ^18^ determination of RNA‐synthesis rates on the basis of single genes,^17^, ^18^ as well as transcriptome‐wide,^19^, ^20^ transcript‐specific RNA turnover mediated by post‐transcriptional gene regulation by microRNAs and *N* ^6^‐methyladenosine,^19^ identification of direct transcription‐factor targets,^21^ analysis of new RNAs after heat shock,^20^ or cellular response to virus infection at the single‐cell level.^22^

These new developments have demonstrated the power of chemical nucleoside conversion to study cellular RNA dynamics upon RNA‐seq.^12^, ^21^, ^22^, ^23^, ^24^, ^25^ Currently, 4sU is the dominantly used nucleoside for metabolic labeling. To enable a greater range of applications, however, an expansion of conversion tools to nucleosides other than 4sU would be highly desirable. In particular, labels that make dual‐labeling approaches possible would significantly increase the flexibility and accuracy of the measurements and they would allow the simultaneous monitoring of RNA processes in different time frames.

**Figure 1:**
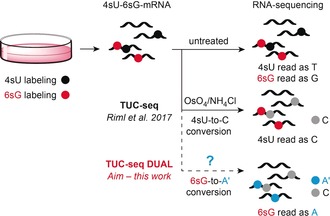
Basic concept of TUC‐seq DUAL. In this work we set out to develop a pair of nucleosides for metabolic double labeling and the required nucleoside conversion chemistry to enable the analysis of mRNA lifetimes upon RNA‐sequencing.

Under these prospects, we set out to develop a concept for dual metabolic labeling and appropriate conversion chemistry for RNA‐seq applications. We envisaged purine nucleoside transformations with the aim to generate apparent G‐to‐A mutations (Figure 1). Such an experimental design should be easily adaptable to the existing 4sU‐based sequencing approaches because handling of complementary U‐to‐C and G‐to‐A data sets in bioinformatics is expected to be straightforward.

## Results and Discussion

**Figure 2:**
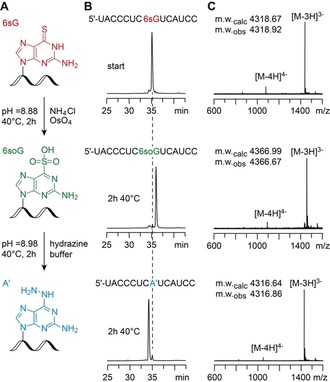
Transformation of 6‐thioguanine (6sG) to 6‐hydrazino‐2‐aminopurine (A′) in a short RNA. A) Chemical structures and reaction conditions; B) Reaction progress followed by anion‐exchange chromatography; C) Verification of the molecular weights of starting material, intermediate, and product RNA by LC‐ESI mass spectrometry.

We selected 6‐thioguanosine (6sG) as a potential convertible nucleoside in RNA. Like 4sU, 6sG is amenable to metabolic labeling.^26^ Thus far and most prominently, 6sG (besides 4sU) has been applied in photoactivatable‐ribonucleoside‐enhanced crosslinking and immunoprecipitation (PAR‐CLIP),^27^, ^28^ a method for high‐resolution mapping of RNA‐binding proteins and their RNA target sites that relies on the photo‐crosslinking reactivity of 6sG and 4sU. Here, we examined the potential of 6sG‐modified RNA for oxidative substitution chemistry. We assumed that the conditions that we previously elaborated for quantitative 4sU‐to‐C conversions (TUC‐seq),^17^, ^18^ namely the application of osmium tetroxide in ammonium chloride buffer, would result in the formation of 2‐aminoadenosine, which should be decoded as adenine in RNA‐sequencing experiments. Hence, we incubated chemically synthesized 6sG‐containing short RNAs with OsO_4_/NH_4_Cl at pH 8.9 for 2 hours at 40 °C (Figure 2). HPLC analysis indicated efficient conversion, however, mass spectrometry revealed that the obtained product was the oxidized RNA species, carrying a sulfonium moiety at C6 (6soG). Harsher conditions using elevated concentrations of NH_4_Cl and/or OsO_4_, and higher reaction temperatures only resulted in partial conversion into the desired 2‐aminoadenosine and besides led to significant degradation of the RNA. Furthermore, replacing ammonium chloride by more reactive hydroxylamine, hydrazine, or *N*‐methylhydrazine solutions did not result in the desired substitutions as major products (Supporting Information, Figure S1). Similarly, 2,2,2‐trifluoroethylamine (TFEA) used previously in TimeLapse‐seq experimentations as the nucleophile,^20^, ^29^ gave either no or only incomplete conversion in our hands (Supporting Information, Figure S1C).

We found, however, that the 6soG‐modified RNA generated by the original TUC‐seq reagent was rather stable and easily isolated by centrifugation using Vivaspin concentrator spin columns. When the oxidized RNA was then treated with buffered hydrazine solution (pH 8.98) at 40 °C for 2 hours, we observed quantitative substitution of the SO_3_^−^ moiety into 6‐hydrazino‐2‐aminopurine (A′). Importantly, this two‐step procedure was mild enough to leave the RNA intact (Figure 2 and Supporting Information, Figure S1). Furthermore, we note that there is no indication for oxidation of the 5,6‐double bond of pyrimidines under the conditions used. This could however become a potential side reaction if higher concentrations of OsO_4_ and additives, such as pyridines or bipyridines, were applied that stabilize the pyrimidine OsO_4_ adducts.^30^, ^31^, ^32^, ^33^

**Figure 3:**
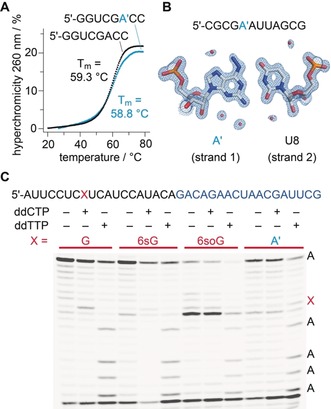
Characterization of 6‐hydrazino‐2‐aminopurine (A′) nucleobases in short RNAs. A) Comparison of melting profiles of unmodified versus A′‐modified palindromic duplexes (RNA sequences as indicated); B) View of the A′–U base pair observed in the crystal structure of a short A′‐modified palindromic duplex at 1.0 Å resolution (RNA sequence as indicated). The 2 *F*_o_−*F*_c_ electron density map contoured at the 1.6 *σ* level is shown in light blue; water molecules are shown as red spheres (PDB code 6XUS); C) Typical gel of a primer extension assay using the RNA as indicated (primer sequence in blue) and Superscript III reverse transcriptase; lanes from left to right are grouped into G, 6sG, 6soG, and A′ templates (red lines), with corresponding C ladders (lanes 2, 5, 8, 11) and T ladders (lanes 3, 6, 9, 12).

Encouraged by these findings, we thoroughly characterized the novel A′ modification. As expected, the thermodynamic stability of an A′–U base pair was comparable to native A–U base pairs in short RNA oligonucleotides (Figure 3 A and Supporting Information, Figure S2). We also analyzed the impact of the precursors 6sG and 6soG on thermodynamic stabilities. These modifications resulted in significantly decreased melting temperatures of double helical oligonucleotides (Supporting Information, Figure S2; for 6sG see also ref. 34). Furthermore, when we crystallized a short palindromic RNA duplex containing the A′ modification and solved its crystal structure at 1.0 Å resolution (Supporting Information, Figure S3 and Table S1), we found that the Watson–Crick mode for A′ pairing with U and the double helical geometry were retained (Figure 3 B and Supporting Information, Figure S3), as encountered in the native duplex.^35^

Next, we tested the impact of the modifications on reverse transcription in a primer extension assay using Superscript III reverse transcriptase (Figure 3 C). Although compared to native guanosine, 6sG caused slightly enhanced termination, the dominant product was the full‐length RNA with 6sG being decoded as G, as judged by the ddCTP sequencing lane (Figure 3 C). Likewise, the vast majority of A′‐containing RNA was reverse transcribed to the full‐length product and only a slight increase in termination was observed. As expected, A′ was read as A (see ddTTP sequencing lane, Figure 3 C). In contrast, for 6soG, the presence of the sulfonic acid group induced almost complete termination (likely because of its bulkiness and/or negative charge) with only minute amounts of full‐length product, in which 6soG was decoded as G (Figure 3 C).

Next, we examined if 6sG is also cleanly converted to A′ under our reaction conditions when it resides in a longer and structured RNA. To this end, we tested a 47 nt RNA oligonucleotide as well as a synthetically generated 76 nt tRNA (*Escherichia coli* tRNA^Phe^) both containing a single 6sG (Supporting Information, Figure S4). Detection of G‐to‐A conversion was performed by reverse‐transcription PCR (RT‐PCR) followed by subcloning and Sanger sequencing. Indeed, for both RNAs, more than 95 % of the original 6sG nucleosides were decoded as A (Supporting Information, Figure S4). Notably, when the same RNAs were treated with OsO_4_/NH_4_Cl only, the majority of 6sG sites were detected as G (Supporting Information, Figure S4), which can be explained by biased PCR amplification of unconverted 6sG‐containing molecules due to transcription termination of 6soG‐containing RNAs, as shown in Figure 3 C. Taken together, these results demonstrate that the new procedure can be successfully applied to long and structured RNA molecules labeled with 6sG.

We then investigated the suitability of 6sG for metabolic labeling. Although cells can take‐up 6sG and incorporate it into RNA and DNA,^26^ conflicting results exist regarding the potential negative effects of 6sG on cell physiology.^26^, ^27^, ^28^, ^36^, ^37^, ^38^ Therefore, we tested the impact of different concentrations and labeling times on cell proliferation in HEK293T cells. Cells were treated with 25, 50, and 100 μm 6sG for 30 and 60 min before the labeling medium was removed, and cells were further cultured for 24, 48, and 72 h (Supporting Information, Figure S5 A). We found that 25 and 50 μm 6sG in the labeling medium caused only a mild reduction in cell numbers at 72 h after labeling for 30 min and slightly stronger inhibition of proliferation when the labeling duration was 60 min. By contrast, 100 μm 6sG had a more pronounced negative effect even at the shorter labeling time (Supporting Information, Figure S5A). Analysis of mRNA expression patterns of selected cell‐cycle‐related genes after a 60 min labeling period with 100 μm 6sG, however, did not reveal perturbed transcript levels (Supporting Information, Figure S5 B).

Because 6sG has reduced base‐pairing strength with cytidine (Supporting Information, Figure S2) and might therefore be less frequently incorporated during transcription in the cell, we next determined whether it is detectable in cellular RNA. Cells were labeled for 2 h with 100 μm 6sG, mRNA was isolated, digested to mononucleosides, and analyzed by RP‐HPLC along with nucleoside standards (Supporting Information, Figure S5 C). We found a clear peak of 6sG in mRNA samples (Supporting Information, Figure S5 C, right panel) that was absent in mRNA from unlabeled cells (Supporting Information, Figure S5 C, middle panel) indicating successful incorporation. Finally, we examined whether OsO_4_‐hydrazine conversion treatment affects the integrity of cellular mRNA preparations. We observed only moderate RNA degradation in converted samples in comparison to untreated mRNA samples (Supporting Information, Figure S5 D,E). Altogether, the results suggest that 6sG is a suitable nucleoside for metabolic labeling experiments provided low concentrations and short labeling times are used.

**Figure 4:**
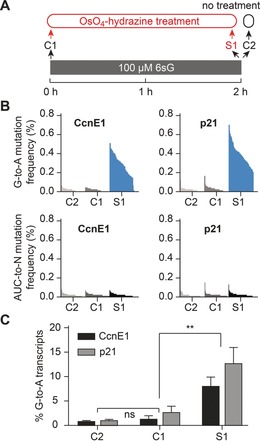
OsO_4_‐hydrazine chemistry allows for efficient detection of new transcripts in pulse‐labeled samples by RNA sequencing. A) Schematics of experimental design. B) Graphs on top: G‐to‐A mutation frequencies for individual G positions in amplicon sequencing reads of the CcnE1 and p21 in the indicated samples. Each G position for which an A exchange was observed is shown as a vertical line. Because labeling occurs randomly, there are different numbers of lines present. Converted Gs are ordered according to their mutation frequency in a descending manner. Graphs on bottom: Same as for graphs on top, except that background mutation frequencies of A, C, and U into any nucleotide (ACU‐to‐N) are shown. C) Relative contribution of labeled transcripts to the total pool of the CcnE1 and p21 transcripts. Mean values±SEM of three biological replicates are shown. Statistical significance was determined by unpaired t‐test (ns, not significant; ** *p*<0.01).

Next, we examined transcript‐specific incorporation and detection of 6sG by OsO_4_‐hydrazine chemistry using the endogenous mRNAs cyclin E1 (CcnE1) and p21, which we had previously used in our TUC‐seq experiments with 4sU, as examples. HEK293T cells were exposed to 100 μm 6sG for two hours before the mRNA was isolated, subjected to OsO_4_‐hydrazine treatment, and subsequently processed by cDNA synthesis and deep sequencing of CcnE1 and p21 PCR amplicons. As controls, we sequenced OsO_4_‐hydrazine‐treated mRNA from unlabeled cells as well as untreated mRNA from cells that were 6sG labeled (controls C1, C2; Figure 4 A). We found clearly increased G‐to‐A mutation frequencies across both amplicons (CcnE1 and p21) in the 6sG‐labeled and OsO_4_‐hydrazine‐treated samples when compared to either control (Figure 4 B, upper panels; Chi‐Square test, p<10^−10^). Importantly, very low mutation frequencies of A, C, and U nucleotides with no differences between test and control samples were detected (Figure 4 B, lower panels). To determine the fraction of new transcripts in the pool of preexisting transcripts, we calculated the percentage of sequencing reads containing at least one G‐to‐A mutation in the test sample (S2) and in the two control samples. In the latter, we found 1–4 % reads with unspecific G‐to‐A mutations, which are most likely caused by PCR or sequencing errors as they occur regardless of whether the cells were labeled with 6sG or whether the RNA was treated with OsO_4_‐hydrazine (Figure 4 C). In the 6sG‐labeled and OsO_4_‐hydrazine‐treated test sample, the fraction of mutated reads was 7.5 % for CcnE1 and approximately 12 % for p21 and therefore significantly above background mutations (Figure 4 C). When corrected for the background mutation rate obtained from sample C1, the fraction of transcripts newly synthesized during the labeling period amounted to 6.5 % for CcnE1 and 10 % for p21. Collectively, the results demonstrate that 6sG incorporation into nascent RNA can be clearly detected by G‐to‐A mutations introduced by OsO_4_‐hydrazine conversion chemistry.

The power of OsO_4_‐hydrazine conversion chemistry resides in its applicability to both 6sG as well as 4sU, thereby allowing simultaneous and efficient processing of 4sU and 6sG double‐labeled RNA. To demonstrate this potential, we set out to determine mRNA lifetimes by applying a 6sG‐4sU double labeling approach. Its advantage over a single‐labeling strategy is illustrated by the following example. When cells are pulse‐labeled with a single modified nucleoside, such as 4sU, followed by a chase period in the presence of excess unlabeled nucleosides, accurate measurement of decay rates is confounded by the fact that a pool of labeled nucleotides remains inside the cell that can still be used for transcriptional incorporation during the chase period. Moreover, internal recycling of labeled nucleotides can additionally interfere.^39^, ^40^ Indeed, the analysis of RNA sampled at different time points during the chase period by TUC‐seq, revealed an apparent ongoing increase of the portion of labeled transcripts (Supporting Information, Figure S6). Hence, transcription and decay cannot be accurately separated. We therefore reasoned that the application of a second complementary metabolic nucleoside label (such as 6sG beside 4sU) will enable us to clearly distinguish transcripts synthesized during the labeling period from those synthesized during the chase period.

**Figure 5:**
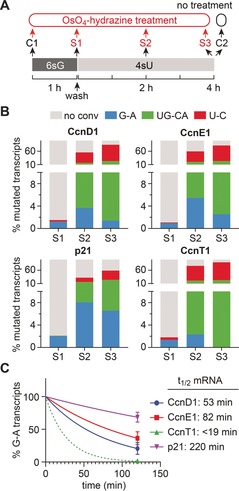
Double metabolic labeling using 6sG and 4sU enables mRNA‐lifetime determination by RNA sequencing (TUC‐seq DUAL). A) Schematics of experimental design. B) Relative contribution of single‐ (blue or red) and double‐labeled (green) transcripts to the total pool of the indicated transcripts. Values were corrected for random mutation using values derived from the unlabeled (no 6sG, C1) samples. C) One‐phase decay plot of transcripts containing G‐A mutations for samples S2 and S3. Values were normalized to the respective S2 values. Mean±SD of three independent experiments is shown. mRNA half‐lives were calculated using the one‐phase‐decay function of GraphPad Prism 7.0.

To test this, we performed a two‐step labeling experiment, in which we first incubated the cells with 100 μm 6sG for 1 h followed by wash‐out of 6sG and addition of labeling medium containing 50 μm 4sU. Samples were collected at different time points after the 6sG pulse (Figure 5 A) and the mRNAs encoding Cyclins D1, E1, and T1 (CcnD1, CcnE1, CcnT1) as well as p21 were analyzed by OsO_4_‐hydrazine treatment and amplicon sequencing. We expected to observe four different types of transcripts: i) transcripts containing 6sG that were synthesized during the initial 6sG pulse, ii) transcripts containing 6sG and 4sU transcribed in the early phase after 6sG wash‐out and 4sU addition, iii) transcripts containing only 4sU that were generated during later time points of 4sU incubation, and iv) unlabeled transcripts corresponding to the preexisting pool.

Indeed, bioinformatic analysis revealed that all four expected transcript types could be detected simultaneously based on increased G‐to‐A and U‐to‐C mutation frequencies (Figure 5 B). We also found that the distribution of the portions of the four different transcript types over time matched their predicted dynamics for the later time points of 4sU labeling (S2 and S3 in Figure 5 A,B): G‐to‐A containing transcripts decreased over time, and UG‐to‐CA as well as U‐to‐C containing transcripts increased at the later time points (Figure 5 B). By contrast, the levels of transcripts containing only G‐to‐A mutations were higher at 2 h (S2 in Figure 5 A,B) after removal of 6sG compared to samples collected right after 6sG labeling (S1 in Figure 5 A,B). This phenomenon is most likely due to initially delayed bioavailability of 4sU nucleotides for incorporation. During the time it takes for 4sU to be metabolized to 4sUTP, remaining 6sGTP inside the cell continues to be incorporated into nascent transcripts. Therefore, we used G‐to‐A transcript levels from S2 and S3 (2 and 4 h after 4sU addition, respectively; Figure 5 A,B) to unambiguously determine mRNA decay rates, since at these time points all newly synthesized transcripts will contain 4sU/6sG or 4sU only. We found the cell‐cycle inhibitor p21 to have the longest lifetime, followed by CcnE1 and CcnD1. The general transcription factor TFIIH subunit CcnT1 exhibited very fast turnover that could not be determined precisely because G‐to‐A‐containing CcnT1 transcripts were no longer detected at 4 h after 4sU addition (Figure 5 C). The mRNA half‐life times of our example genes determined by TUC‐seq DUAL are at the lower edge of a broad range of lifetimes observed before in other cell types and using different methodology.^41^, ^42^, ^43^ To resolve these ambiguities, however, direct side‐by‐side comparison of different methods is needed in future studies. We also note that labeling conditions (duration of first pulse, duration of second pulse, sampling times) depend on the cell type, the particular transcript, and the research question in general and therefore need to be optimized for each experiment.

## Conclusion

In this study, we have developed the selective and quantitative conversion of 6sG‐ into A′‐containing RNA, where A′ constitutes a 6‐hydrazino purine derivative that is cleanly decoded as adenosine in RNA sequencing experiments. Because 6sG is applicable to metabolic labeling of freshly synthesized RNA and because the new OsO_4_/NH_4_Cl/hydrazine conversion chemistry is fully compatible with the conversion of the frequently applied metabolic label 4‐thiouridine (4sU) into C (TUC‐seq),^17^, ^18^ the combination of both modified nucleosides in dual labeling setups enables high accuracy measurements of RNA decay. A pulse‐pulse dual labeling strategy minimizes or eliminates two major caveats of currently employed pulse‐chase settings, namely the persistence of the labeled nucleotide in the cellular nucleotide pool after removal of the labeling medium on one hand and the internal recycling of modified nucleotides^39^, ^40^ on the other hand. Both result in incomplete chase and lead to an overestimation of RNA half‐lives. Our method now offers the option to use two modified nucleotides in subsequent pulses and their simultaneous detection. Thus, synthesis and decay of mRNA can be clearly distinguished based on the differential presence of G‐to‐A and U‐to‐C mutations. This enables mRNA lifetime evaluation with unprecedented precision.

## Conflict of interest

R.M., A.L., C.G., I.D. have applied for intellectual property rights for this work.

## Supporting information

As a service to our authors and readers, this journal provides supporting information supplied by the authors. Such materials are peer reviewed and may be re‐organized for online delivery, but are not copy‐edited or typeset. Technical support issues arising from supporting information (other than missing files) should be addressed to the authors.

Supplementary
