# Supplementary material for: Thioguanosine Conversion Enables mRNA‐Lifetime Evaluation by RNA Sequencing Using Double Metabolic Labeling (TUC‐seq DUAL)
Source: Angew Chem Int Ed Engl. 2020 Feb 28;59(17):6881–6. doi: 10.1002/anie.201916272 (PMC7186826; doi:10.1002/anie.201916272)
Supplement: Supplementary file 1 — Supplementary [file ANIE-59-6881-s001.pdf]

Angewandte Chemie
Eine Zeitschrift der Gesellschaft Deutscher Chemiker

Supporting Information

Thioguanosine Conversion Enables mRNA-Lifetime Evaluation by RNA Sequencing Using Double Metabolic Labeling (TUC-seq DUAL)

Catherina Gasser[+], Isabel Delazer[+], Eva Neuner, Katharina Pascher, Karl Brillet, Sarah Klotz, Lukas Trixl, Maximilian Himmelstoß, Eric Ennifar, Dietmar Rieder, Alexandra Lusser,* and Ronald Micura**

anie_201916272_sm_miscellaneous_information.pdf

Contents

1. Supporting Methods 2

1.1. Conversion of 6sG containing RNA 2
1.2. Synthesis of 6-thioguanosine phosphoramidite 3
General 3
Overview of the synthetic route 3
2'-*O*-(*tert*-Butyldimethylsilyl)-3',5'-*O*-(di-*tert*-butylsilylene)guanosine (**2**) 4
N^2-Acetyl-2'-*O*-(*tert*-butyldimethylsilyl)-3',5'-*O*-(di-*tert*-butylsilylene)guanosine (**3**) 5
N^2-Acetyl-2'-*O*-(*tert*-butyldimethylsilyl)-3',5'-*O*-(di-*tert*-butylsilylene)-6-(2-cyanoethylthio)guanosine (**4**) 6
N^2-Acetyl-2'-*O*-(*tert*-butyldimethylsilyl)-6-(2-cyanoethylthio)-guanosine (**5**) 8
N^{2-}Acetyl-2'-*O*-(*tert*-butyldimethylsilyl)-5'-*O*-(4,4'-dimethoxytrityl)-6-(2-cyanoethylthio)guanosine (**6**) 9
N^2-Acetyl-2'-*O*-(*tert*-butyldimethylsilyl)-5'-*O*-(4,4'-dimethoxytrityl)-
6-(2-cyanoethylthio)-guanosine-3'-*O*-2-cyanoethyl-*N,N*-diisopropylphosphoramidite (**7**) 11
1.3. Preparation of RNA 13
1.1.1. Solid-phase synthesis of oligoribonucleotides 13
1.1.2. Deprotection of oligoribonucleotides 13
1.1.3. Purification of oligoribonucleotides 13
1.1.4. Mass spectrometry of oligoribonucleotides 13
1.4. UV melting analysis 14
1.5. Crystallography and X-ray analysis 14
1.6. Primer extension analysis 14
1.7. PCR-mediated detection of 6sG-to-A' conversion 15
1.8. Single and double labeling of HEK293T cells 15
1.9. Proliferation assay 15
1.10. RNA isolation and RT-qPCR analysis 15
1.11. 6sG incorporation analysis by HPLC 15
1.12. Amplicon sequencing and data analysis 16
1.13. Statistical analyses 16

2. Supporting Figures 17

Supporting Figure S1. Optimization of 6sG conversion. 17
Supporting Figure S2. Melting profiles of 6sG, 6soG, and A'-containing RNA and unmodified counterparts 18
Supporting Figure S3. Crystal structure of 5'-CGCGA'AUUAGCG 19
Supporting Figure S4. Conversion of 6sG in structured RNAs 20
Supporting Figure S5. Characterization of suitability of 6sG for metabolic labeling 21
Supporting Figure S6. Ongoing incorporation of 4sU in a pulse-chase labeling experiment 22

3. Supporting Table 23

Supporting Table 1. X-ray data collection and crystallographic refinement statistics 23
Supporting Table 2. List of primer sequences 24

4. Supporting Information References 25

1. Supplementary Methods

1.1. Conversion of 6sG containing RNA

OsO_4 stock solution (100 mM, 1.5 mL; stored at 4 °C in a sealed glass vial) and ammonium chloride buffer (2 M, pH 8.88) were prepared according to references [1,2]. The hydrazine stock solution (1.5 M) was made by adding hydrazine monohydrate (7.51 g, 150 mmol) into 100 mL of an aqueous solution containing tris(hydroxymethyl)aminomethane (Tris base) (1.21 g, 10 mmol; 0.1 M) and ethylenediaminetetraacetic acid (EDTA) (146 mg, 0.5 mmol; 5 mM). The pH was adjusted with concentrated HCl to 8.98. For the conversion reaction, a 1 mM aqueous OsO_4 solution was freshly prepared from the OsO_4 stock solution.

Conversion procedure: Synthetic 6sG containing RNA (1 nmol) was mixed with NH_4Cl buffer (2 µL, 2 M, pH 8.88), OsO_4 solution (10 µL, 1 mM), and H_2O to give final concentrations of 0.45 mM OsO_4 and 180 mM NH_4Cl in a total volume of 22 µL. The mixture was incubated for 2 hours at 40 °C, then transferred into Vivaspin 500 (MWCO 3000, PES) centrifugal concentrators (Sartorius, Göttingen, Germany) and washed two times with 400 µL of water.

Afterwards, the RNA was lyophilized, dissolved in water (15 µL), and hydrazine stock solution (5 µL, 1.5 M, pH 8.98) was added to give a final concentration of 375 mM hydrazine in a total volume of 20 µL. The solution was incubated for 2 hours at 40 °C. The RNA solution was transferred into centrifugal concentrators and washed four times with 400 µL of water as described before. Progress of the RNA conversion reactions was monitored by anion exchange HPLC; 6soG and A' modified RNA was stored at –20 °C in aqueous solution or lyophilized. As an alternative to ultrafiltration, the RNA can also be precipitated by adding 90 µL of precipitation solution (650 µL water, 150 µL 1 M NaOAc, pH 5.2, 10 µL glycogen (20 mg mL^{-1})) and 250 µL of cold ethanol, stored at –20 °C for 30 minutes, followed by centrifugation (13000 rpm, 4 °C, 30 min). The supernatant was discarded and the RNA was lyophilized or dissolved in water (15 µL) and further processed as described above.

For isolated, metabolically labeled cellular RNA, a milder hydrazine stock solution was used. Briefly, after OsO_4/NH_4Cl treatment and precipitation, the RNA pellet was resuspended in water (15 µL) and hydrazine stock solution (5 µL; 0.5 M, pH 8.11, 5 mM EDTA, 0.5 M Tris) was added to give a final concentration of 125 mM hydrazine.

1.2. Synthesis of 6-thioguanosine phosphoramidite

General and synthesis overview. ^{1}H, ^{13}C and ^{31}P NMR spectra were recorded on a Bruker DRX 300 MHz or Avance II+ 600 MHz instrument. The chemical shifts are referenced to the residual proton signal of the deuterated solvents: $CDCl_3$ (7.26 ppm), d_6-DMSO (2.49 ppm) for ^{1}H NMR spectra; $CDCl_3$ (77.0 ppm) or d_6-DMSO (39.5 ppm) for ^{13}C NMR spectra. ^{31}P-shifts are relative to external 85% phosphoric acid. ^{1}H- and ^{13}C-assignments were based on COSY and HSQC experiments. MS experiments were performed on a Finnigan LCQ Advantage MAX ion trap instrument. Analytical thin-layer chromatography (TLC) was carried out on Marchery-Nagel Polygram SIL G/UV254 plates. Flash column chromatography was carried out on silica gel 60 (70-230 mesh). All reactions were carried out under argon atmosphere. Chemical reagents and solvents were purchased from commercial suppliers and used without further purification. Organic solvents for reactions were dried overnight over freshly activated molecular sieves (3 Å).

Overview of the synthetic route toward 6sG

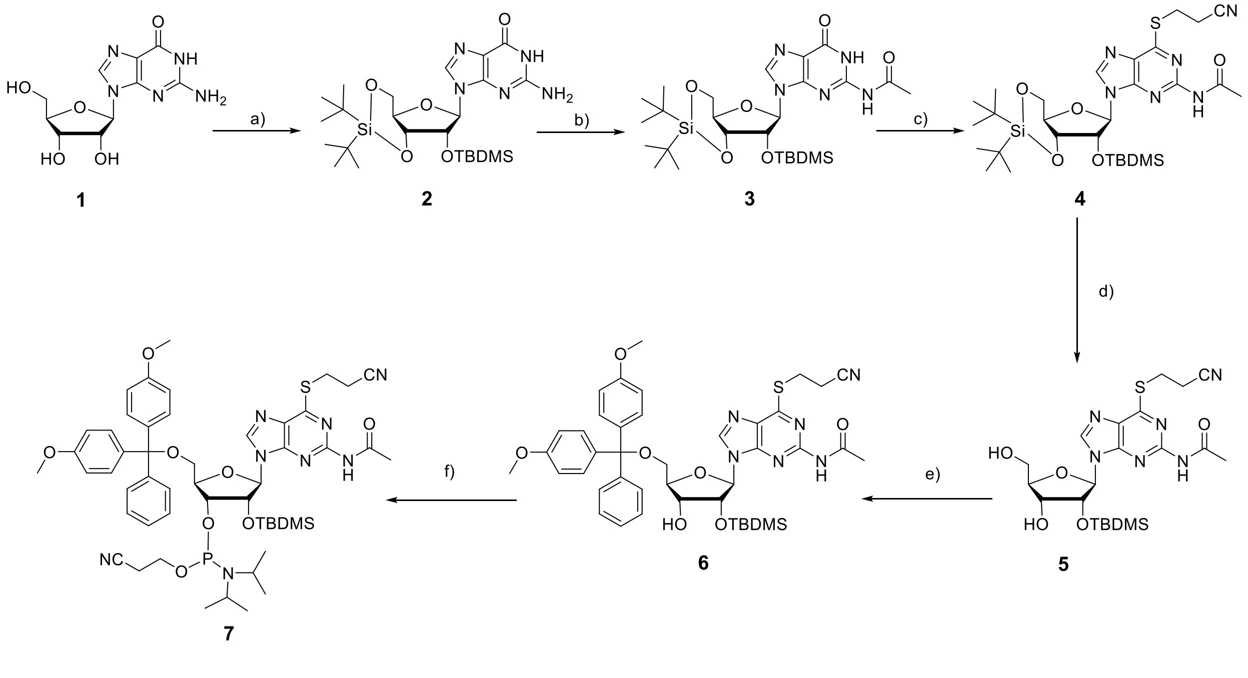

Reaction conditions: a) i. 1.1 eq. $(tBu)_2Si(OTf)_2)$, 30 min, 0 °C, ii. 5 eq. imidazole, 5 min, 0 °C, 25 min, rt, iii. 1.2 eq. TBDMSCl, 2 h, 60 °C, DMF (68%); b) 2 eq. AcCl, 90 min, 0 °C, pyridine, CH_2Cl_2 (84%); c) i. 1.2 eq mesitylenesulfonyl chloride, 4 eq. Et_3N, 0.05 eq. DMAP, 1 h, rt, ii. 10 eq. *N*-methylpyrrolidine, 30 min, 0 °C, iii. 10 eq. 3-mercaptopropionitrile in THF, 30 min, 0 °C, CH_2Cl_2 (93%); d) 3.85 eq. HF-pyridine, 2 h, 0 °C, CH_2Cl_2 (93%); e) 1.3 eq. DMTCl, 18 h, rt, pyridine, CH_2Cl_2 (86%); f) 0.6 eq. *N*-methylimidazole, 7 eq. *sym*-collidine, 2.5 eq. CEPCl, 40 min, rt, THF (61%).

2'-*O*-(*tert*-Butyldimethylsilyl)-3',5'-*O*-(di-*tert*-butylsilylene)guanosine (2)

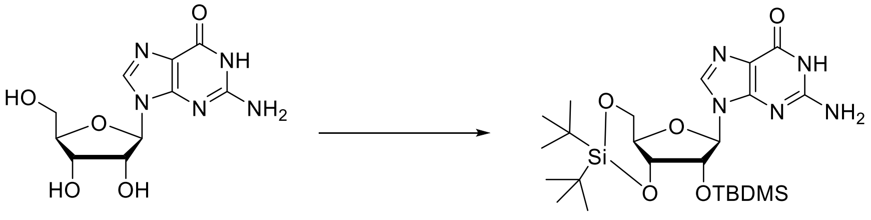

1 $C_{10}H_{13}N_5O_5$ [283.24] → **2** $C_{24}H_{43}N_5O_5Si_2$ [537.81]

Guanosine **1** (150 mg, 0.53 mmol) was co-evaporated twice with pyridine and dried under high vacuum for 30 min. Compound **1** was suspended in dry *N*,*N*-dimethylformamide (1.05 mL), cooled to 0 °C and di-*tert*-butylsilyl bis(trifluoromethanesulfonate) (256 mg, 0.58 mmol, 1.1 eq.) was added dropwise over 10 min. After stirring for 30 min at 0 °C, imidazole (180 mg, 2.65 mmol, 5 eq.) was added and stirred for 5 min at 0 °C and 25 min at room temperature. Subsequently, the solution was treated with *tert*-butyldimethylsilyl chloride (96 mg, 0.64 mmol, 1.2 eq.) and stirred two hours at 60 °C. After cooling to room temperature the suspension was filtrated and washed with cold methanol. The precipitant (**2**) was dried under high vacuum. Yield: 195 mg white powder (68%). ^{1}H-NMR in d$_6$-DMSO (300 MHz): δ 10.61 (s, 1H, N(1)*H*); 7.88 (s, 1H, C(8)*H*); 6.31 (s, 2H, N*H$_2$*); 5.69 (s, 1H, *H*(1')); 4.54 (d, J= 5.10 Hz, 1H, *H*(2')); 4.32–4.24 (m, 2H, *H*(3'), *H*(4')); 3.95–3.90 (m, 2H, *H$_2$*(5')); 1.04, 0.98, 0.84 (3s, 27H, Si(C(C*H$_3$*)$_3$)$_2$, SiC(C*H$_3$*)$_3$); 0.07, 0.04 (s, 6H, Si(C*H$_3$*)$_2$) ppm.

^{1}H NMR (300 MHz, DMSO-d$_6$) spectrum of compound **2**:

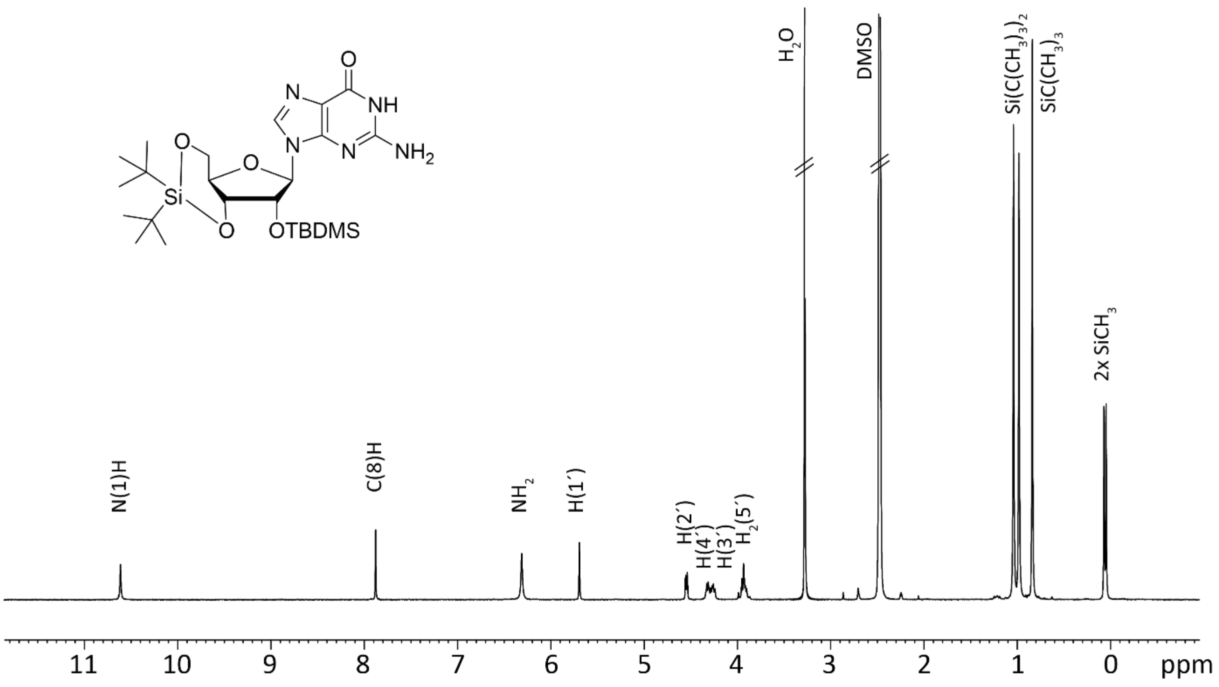

***N*²-Acetyl-2'-*O*-(*tert*-butyldimethylsilyl)-3',5'-*O*-(di-*tert*-butylsilylene)guanosine (3)**

Compound **2** (195 mg, 0.36 mmol) was suspended in dichloromethane (5.0 mL) and pyridine (1.8 mL) and cooled to 0 °C. Acetyl chloride (57 mg, 0.73 mmol, 2 eq.) was added dropwise over a period of 10 min. After stirring for 90 min at 0 °C the solvent was evaporated and the orange solid was purified by column chromatography (SiO_2, 2-3% (v/v) methanol/ dichloromethane). Yield: 175 mg white foam (84%). TLC: 5% (v/v) methanol/ dichloromethane: R_f= 0.44. ^{1}H NMR in $CDCl_3$ (300 MHz): δ 12.04 (s, 1H, N(1)*H*); 8.96 (s, 1H, C(2)N*H*); 7.68 (s, 1H, C(8)*H*); 5.79 (s, 1H, *H*(1')); 4.49–4.44 (m, 1H, *H*(4')); 4.36 (d, J = 4.29 Hz, 1H, *H*(2')); 4.27–4.12 (m, 2H, *H*(3'), *H*(5')); 4.01–3.94 (m, 1H, *H*(5')); 2.30 (s, 3H, C(O)CH_3); 1.06, 1.02, 0.87 (3s, 27H, Si(C(CH_3)$_3$)$_2$, SiC(CH_3)$_3$); 0.10, 0.09 (s, 6H, Si(CH_3)$_2$) ppm.

^{1}H NMR (300 MHz, $CDCl_3$) spectrum of compound **3**:

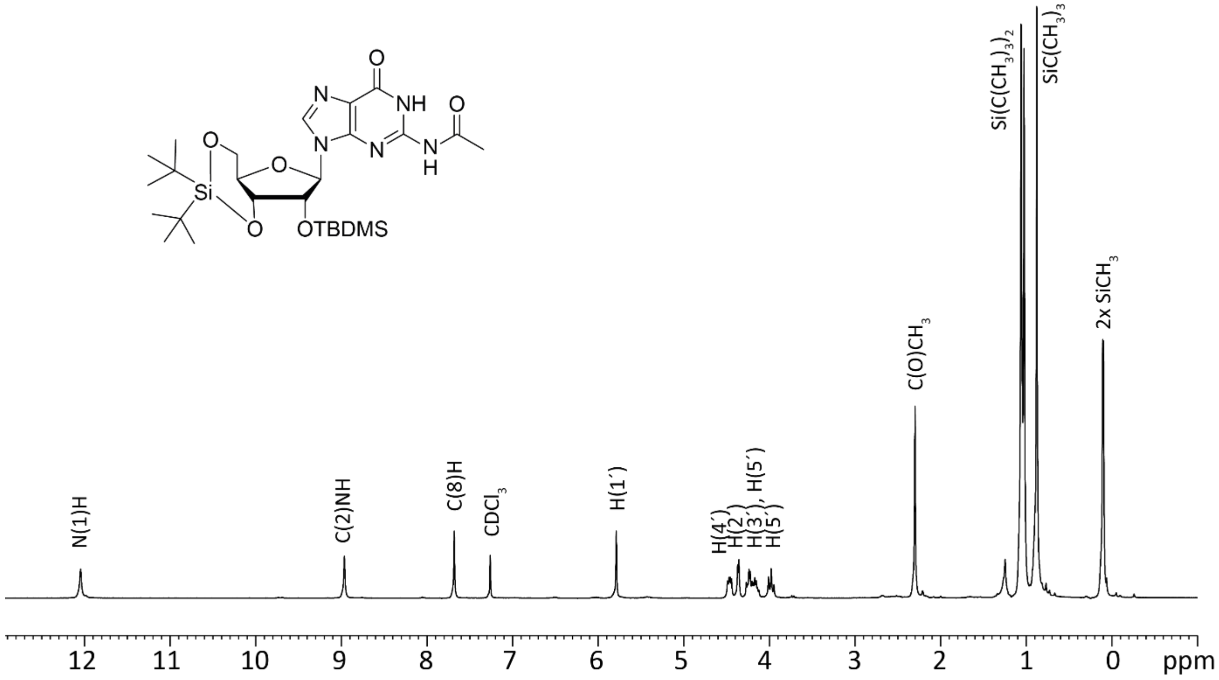

N^2-Acetyl-2'-*O*-(*tert*-butyldimethylsilyl)-3',5'-*O*-(di-*tert*-butylsilylene)-6-(2-cyanoethylthio)-guanosine (4)

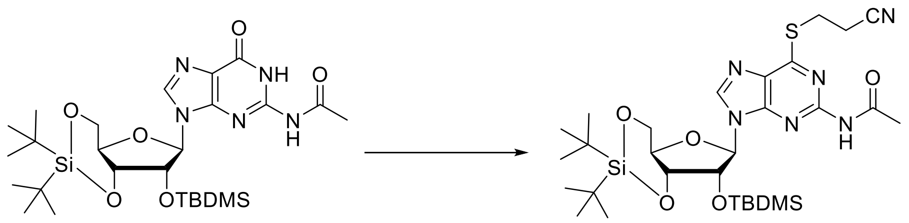

3
$C_{26}H_{45}N_5O_6Si_2$
[579.85]

4
$C_{29}H_{48}N_6O_5SSi_2$
[648.97]

3-Mercaptopropionitrile was prepared freshly by placing sodium borohydride (341 mg, 9.00 mmol, 17 eq.) in a 100 mL round bottom flask and depleting it from oxygen under high vacuum for 15 min. Then dry tetrahydrofuran (11 mL) and 3,3'-dithiobis(propionitrile) (445 mg, 2.59 mmol, 5 eq.) were added. Dry ethanol was added dropwise until gas formation was observed. In the meantime, compound **3** (300 mg, 0.52 mmol) was dissolved in dichloromethane (5.5 mL), mesitylene-2-sulfonyl chloride (136 mg, 0.62 mmol, 1.2 eq.), triethylamine (211 mg, 2.07 mmol, 4 eq.) and 4-(dimethylamino)pyridine (3.6 mg, 0.03 mmol, 0.05 eq.) were added and stirred at room temperature for 90 min. After cooling to 0 °C, *N*-methylpyrrolidine (440 mg, 5.17 mmol, 10 eq.) was added and stirring was continued for 30 min at 0 °C. The previously prepared 3-mercaptopropionitrile was added via a syringe to the solution at 0 °C. After 45 min, the mixture was washed twice with 1 M KH_2PO_4 solution, dried over Na_2SO_4 and evaporated. The residue was purified by column chromatography (SiO_2, 1-1.75% (v/v) methanol/dichloromethane) to obtain compound **4**. Yield: 316 mg white foam (93%). TLC: 3% (v/v) methanol/dichloromethane: R_f = 0.41. ^{1}H-NMR in $CDCl_3$ (300 MHz): δ 7.90 (s, 1H, C(2)N*H*); 7.87 (s, 1H, C(8)*H*); 5.88 (s, 1H, *H*(1')); 4.56 (d, J = 4.67 Hz, 1H, *H*(2')); 4.48 (m, 1H, *H*(5')); 4.35 (m, 1H, *H*(3')); 4.21 (m, 1H, *H*(4')); 3.99 (t, J = 9.74 Hz, 1H, *H*(5')); 3.57 (t, J = 7.07 Hz, 2H, SC*H*$_2$); 2.99 (t, J = 7.00, 2H, C*H*$_2$CN); 2.49 (s, 3H, C(O)C*H*$_3$); 1.09, 1.05, 0.93 (3s, 27H, Si(C(C*H*$_3$)$_3$)$_2$, SiC(C*H*$_3$)$_3$); 0.14 (s, 6H, Si(C*H*$_3$)$_2$) ppm. ^{13}C-NMR in $CDCl_3$ (75 MHz): δ 141.1 (C(8)); 92.4 (C(1')); 76.4 (C(3')); 75.6 (C(2')); 74.8 (C(4')); 67.9 (C(5')); 27.6, 27.1, 26.0 (Si(C(*C*H$_3$)$_3$)$_2$, SiC(*C*H$_3$)$_3$); 25.2 (C(O)*C*H$_3$); 24.9 (S*C*H$_2$); 20.5 (*C*H$_2$CN); -4.4, -4.8 (2x $SiCH_3$) ppm. ESI-MS: $[M+H]^+$ calculated for $C_{29}H_{49}N_6O_5SSi_2^+$ 649.30, found 649.26.

^1H NMR (300 MHz, $CDCl_3$) spectrum of compound **4**:

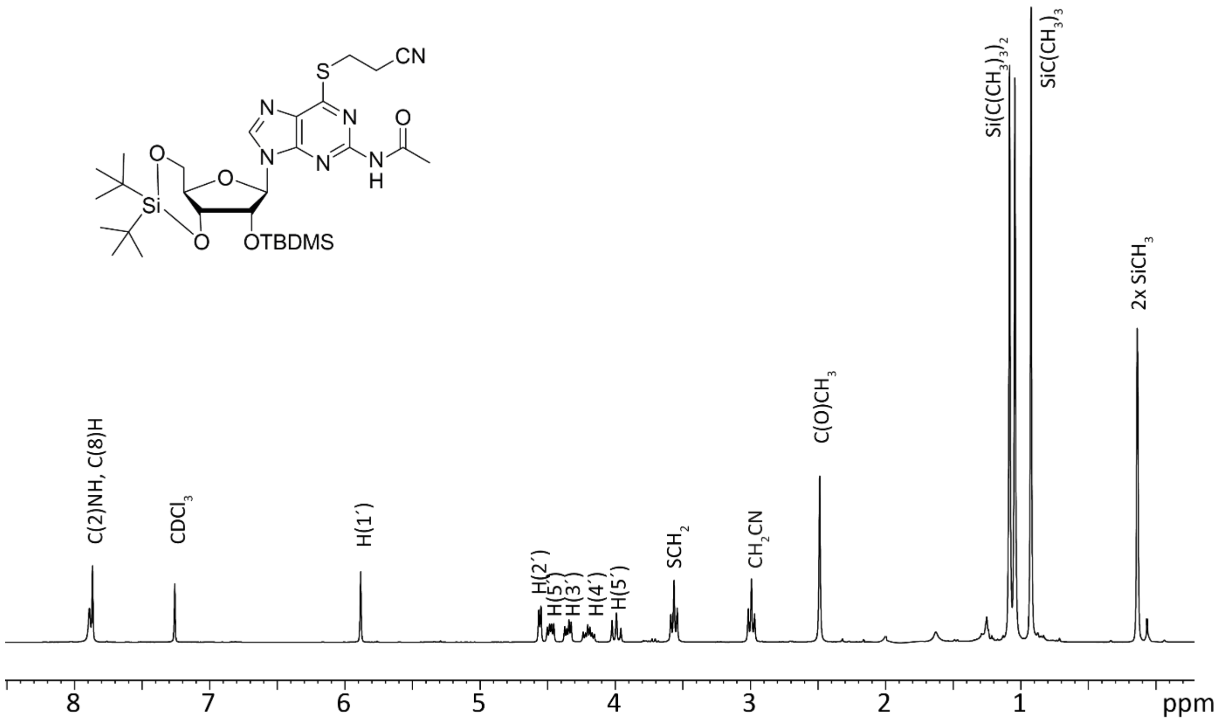

^{13}C NMR (75 MHz, $CDCl_3$) spectrum of compound **4**:

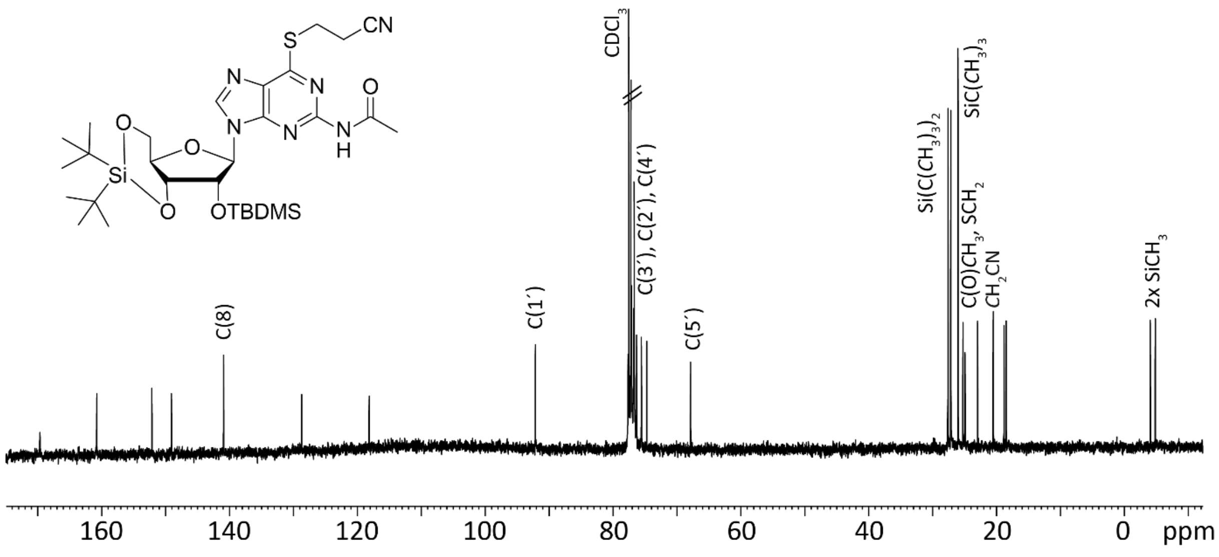

N[2]-Acetyl-2'-*O*-(*tert*-butyldimethylsilyl)-6-(2-cyanoethylthio)-guanosine (5)

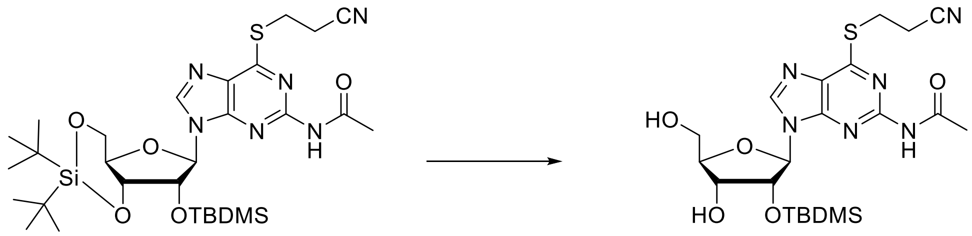

**4**	**5**
$C_{29}H_{48}N_6O_5SSi_2$	$C_{21}H_{32}N_6O_5SSi$
[648.97]	[508.67]

Hydrogen fluoride in pyridine (Sigma Aldrich, 8 M, 174 µl, 6.88 mmol, 3.85 eq) was diluted under cooling with pyridine (1.08 mL) and added to a solution of compound **4** (1.16 g, 1.79 mmol) in dichloromethane (8.7 mL) at 0 °C. The solution was stirred for two hours at 0 °C, washed once with water and twice with saturated aqueous bicarbonate, dried over Na_2SO_4 and then evaporated. Product **5** as white foam was used in the next step without further purification. For NMR spectra the product was purified by column chromatography (SiO_2, 1-3% (v/v) methanol/dichloromethane). Yield: 846 mg white foam (93%). TLC: 5% (v/v) methanol/dichloromethane: R_f = 0.31. ^{1}H NMR in $CDCl_3$ (300 MHz): δ 8.16 (s, 1H, C(2)N*H*); 7.95 (s, 1H, C(8)*H*); 5.77 (d, J = 6.72 Hz, 1H, *H*(1')); 4.99 (t, J = 5.83 Hz, 1H, *H*(2')); 4.38 (m, 1H, *H*(3')); 4.27 (m, 1H, *H*(4')); 3.98–3.75 (m, 2H, *H*(5')); 3.62–3.48 (m, 2H, SC*H*$_2$); 3.00 (t, J = 7.00, 2H, C*H*$_2$CN); 2.79 (s, 1H, C(3´)O*H*); 2.34 (s,3H,C(O)C*H*$_3$); 0.80 (s, 9H, SiC(C*H*$_3$)$_3$); -0.12, -0.32 (2s, 6H, 2x SiC*H*$_3$) ppm. ^{13}C-NMR in $CDCl_3$ (75 MHz): δ 143.3 (C(8)); 91.1 (C(1')); 87.3 (C(4')); 75.2 (C(2')); 72.5 (C(3')); 63.0 (C(5')); 26.1 (SiC(*C*H$_3$)$_3$); 25.5 (C(O)*C*H$_3$); 25.3 (S*C*H$_2$); 19.1 (*C*H$_2$CN); -4.8 (2x SiCH$_3$) ppm. ESI-MS: $[M+H]^+$ calculated for $C_{21}H_{32}N_6O_5SSi$ 509.20, found 509.18.

^{1}H NMR (300 MHz, $CDCl_3$) spectrum of compound **5**:

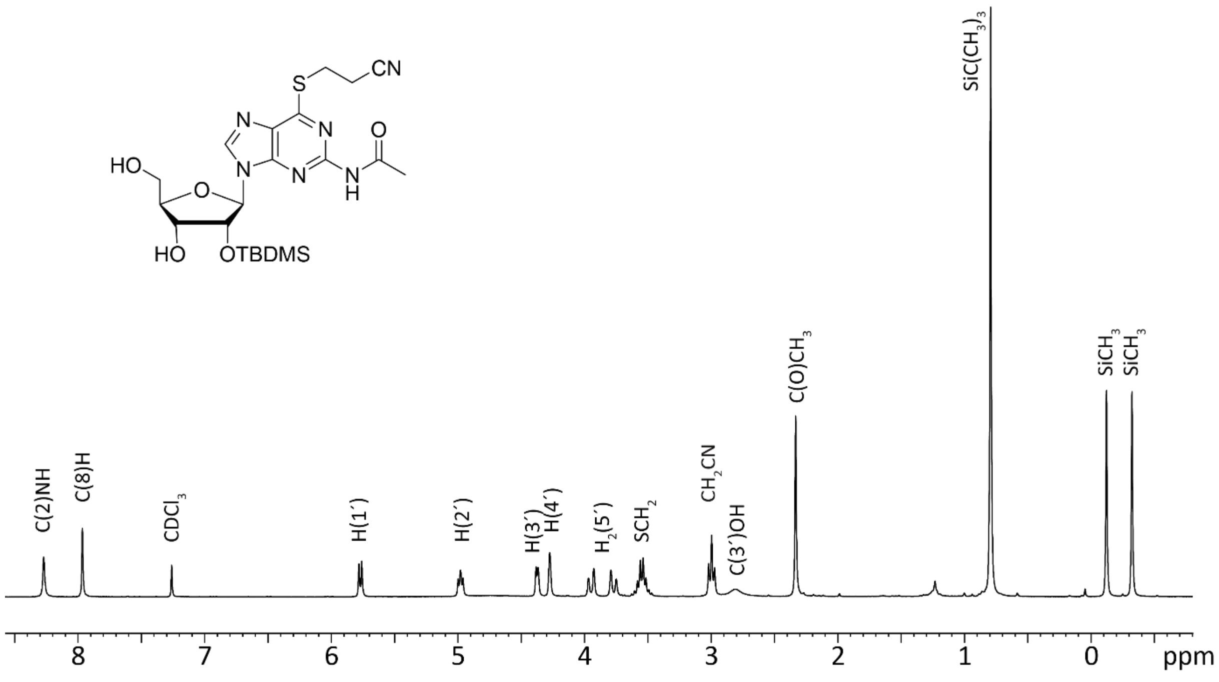

^{13}C NMR (75 MHz, $CDCl_3$) spectrum of compound **5**:

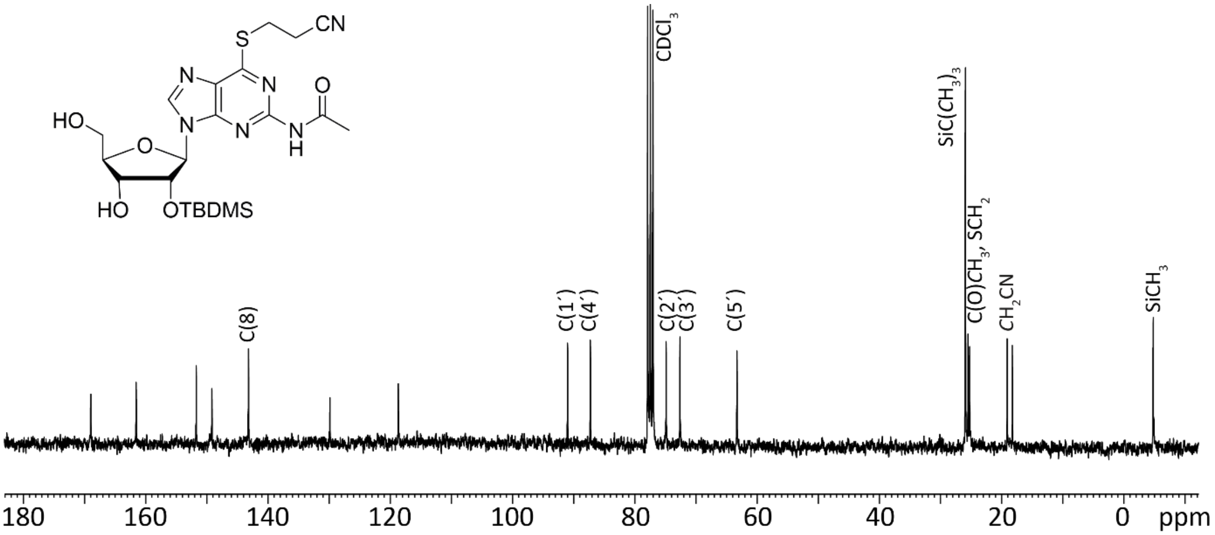

N^{2}-Acetyl-2'-*O*-(*tert*-butyldimethylsilyl)-5'-*O*-(4,4'-dimethoxytrityl)-6-(2-cyanoethylthio)-guanosine (6)

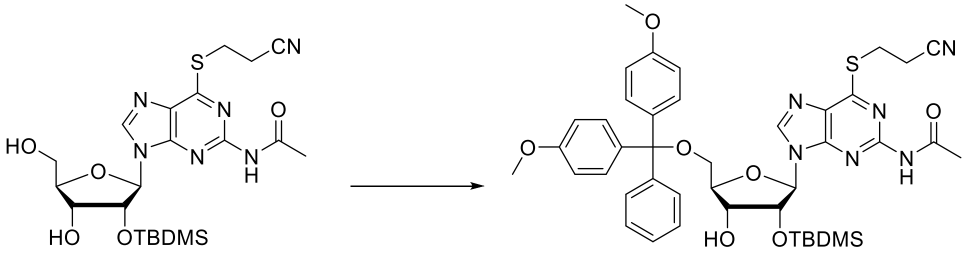

5
$C_{21}H_{32}N_6O_5SSi$
[508.67]

6
$C_{42}H_{50}N_6O_7SSi$
[811.04]

Compound **5** (611 mg, 1.20 mmol) was co-evaporated three times with pyridine and dried under high vacuum for 30 min. 4,4´-Dimethoxytrityl chloride (529 mg, 1.56 mmol, 1.3 eq.) was added to a solution of compound **5** in pyridine (13 mL) and stirred for 18 hours at room temperature. The solution was concentrated *in vacuo*, diluted with dichloromethane, washed three times with saturated aqueous bicarbonate, dried over Na_2SO_4 and evaporated. The residue was purified by column chromatography (SiO_2, 0.25 -1% (v/v) methanol/dichloromethane/ 1% (v/v) triethylamine) to give compound **6** as white foam. Yield: 837 mg white foam (86%). TLC: 4% (v/v) methanol/dichloromethane: R_f = 0.30. ^{1}H NMR in $CDCl_3$ (300 MHz): δ 8.05 (s, 1H, C(8)*H*); 7.78 (s, 1H, C(2)N*H*); 7.51–7.18 (m, 9H, C(ar)*H*); 6.81–6.77 (m, 4H, C(ar)*H*); 5.92 (d, J = 6.31 Hz, 1H, *H*(1')); 5.09 (t, J = 5.49 Hz, 1H, *H*(2')); 4.48–4.32 (m, 1H, *H*(3')); 4.24 (m, 1H, *H*(4')); 3.78, 3.77 (2s, 6H, 2x OCH_3); 3.56–3.51 (m, 3H, SCH_2, *H*(5')); 3.30–3.25 (m, 1H, *H*(5')); 3.05 (t, J = 6.93, 2H, CH_2CN); 2.75 (d, J = 2.33 Hz, 1H, O*H*); 1.94 (s,3H,C(O)CH_3); 0.83 (s, 9H, SiC(CH_3)$_3$); -0.00, -0.20 (2s, 6H, 2x SiCH_3) ppm. ^{13}C-NMR in $CDCl_3$ (75 MHz): δ 142.1 (C(8)); 130.3–128.3, 113.4 (C(ar)); 88.3 (C(1')); 84.8 (C(4')); 75.1

(C(2')); 71.6 (C(3')); 64.0 (C(5')); 55.4 (2x O*C*H3); 25.7 (SiC(*C*H3)3); 25.2 (C(O)*C*H3); 24.7 (S*C*H2); 19.0 (*C*H2CN); -4.8 (2x SiCH3) ppm. ESI-MS: $[M+Na]^+$ calculated for $C_{42}H_{50}N_6O_7SSi$ 833.31, found 833.27.

^{1}H NMR (300 MHz, $CDCl_3$) spectrum of compound **6**:

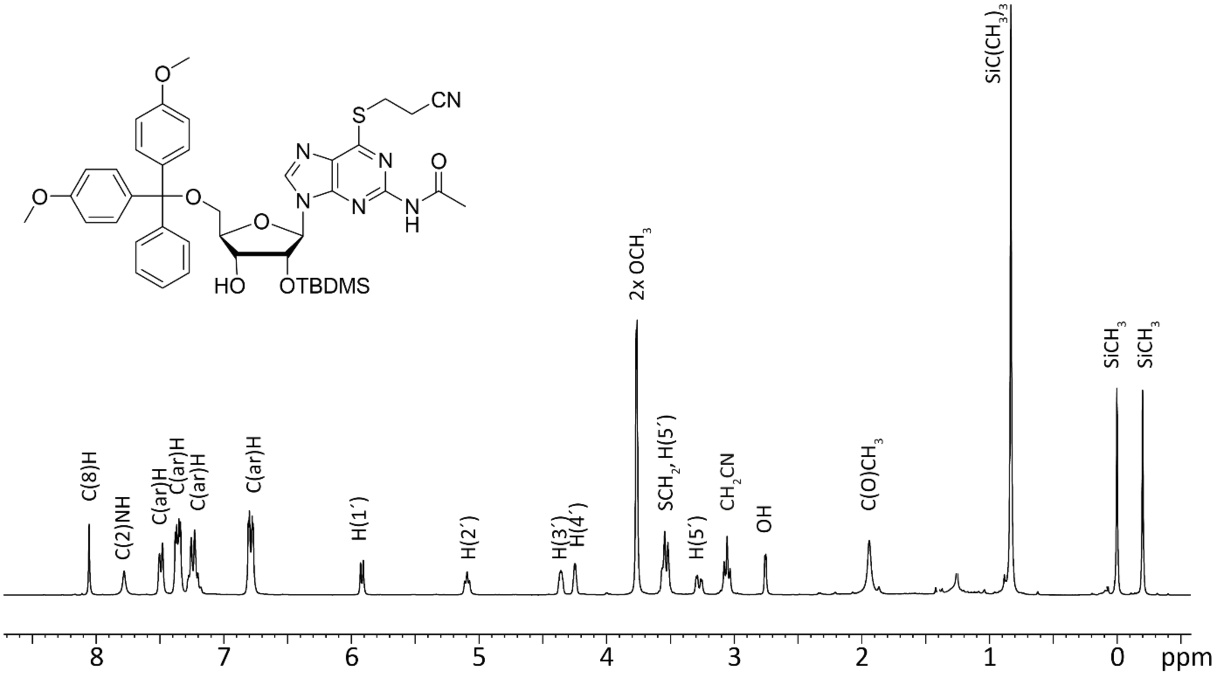

^{13}C NMR (75 MHz, $CDCl_3$) spectrum of compound **6**:

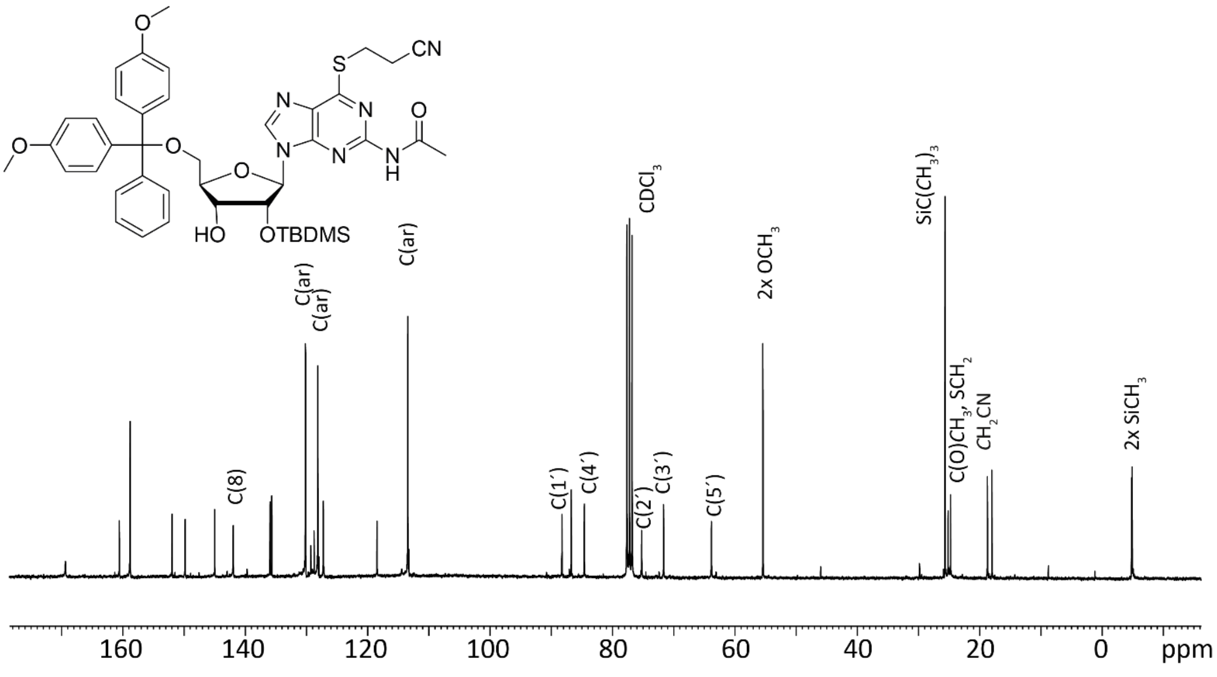

N^2-Acetyl-2'-*O*-(*tert*-butyldimethylsilyl)-5'-*O*-(4,4'-dimethoxytrityl)-6-(2-cyanoethylthio)-guanosine-3'-*O*-2-cyanoethyl-*N,N*-diisopropylphosphoramidite (7)

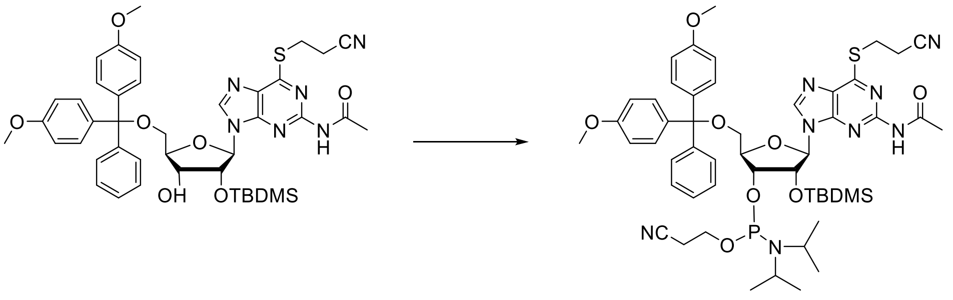

**6**	**7**
$C_{42}H_{50}N_6O_7SSi$	$C_{51}H_{67}N_8O_8PSSi$
[811.04]	[1011.26]

Compound **6** (320 mg, 0.39 mmol) was co-evaporated three times with dry pyridine, three times with dry toluene, three times with dry tetrahydrofuran, and finally dissolved in tetrahydrofuran (1.41 mL). *N*-methylimidazole (20 mg, 0.24 mmol, 0.6 eq.), 2,4,6-trimethylpyridine (342 mg, 2.82 mmol, 7 eq.) and 2-cyanoethyl-*N,N*-diisopropylchlorophosphoramidite (216 mg, 0.94 mmol, 2.5 eq.) were added. After 40 min, the solution was diluted with dichloromethane and washed three times with saturated aqueous bicarbonate solution, dried over Na_2SO_4, filtered and evaporated. The product was purified by column chromatography (10 g SiO_2, 30% (v/v) ethyl acetate/n-hexane/ 1%(v/v) triethylamine). Yield: 242 mg white foam (61%). TLC: 4% (v/v) methanol/ dichloromethane: R_f = 0.31. ^{1}H NMR in $CDCl_3$ (300 MHz): δ 8.09 (s, 1H, C(8)*H*); 7.95 (s, 1H, C(2)N*H*); 7.68 (s, 1H, C(2)N*H*); 7.55–7.21 (m, 9H, C(ar)*H*); 6.83–6.79 (m, 4H, C(ar)*H*), 6.00, 5.90 (d, J = 7.41Hz, 1H, *H*(1')); 5.05 (m, 1H, *H*(2')); 4.40–4.26 (m, 2H, *H*(3'), *H*(4')); 4.07–3.85 (m, 2H, OC*H*2); 3.77 (s, 6H, 2x OC*H*3); 3.65–3.51 (m, 8H, OC*H*2,N(C*H*(CH_3)2)2, SC*H*2, C*H*2CN); 3.24–3.04 (m, 4H, C*H*2CN, *H*2(5´)); 2.70 (t, J = 6.72, 2, C*H*2CN); 2.33–2.13 (m, 2H, C*H*2CN); 1.92, 1.83 (1s,3H,C(O)C*H*3);1.21–0.98 (m, 12H, N(CH(C*H*3)2)2); 0.76 (s, 9H, SiC(C*H*3)3); -0.02, -0.25 (s, 6H, 2x SiC*H*3) ppm. ^{31}P-NMR in $CDCl_3$ (121 MHz): δ 152.8, 149.7 ppm. ESI-MS: $[M+H+Et_3N]^+$ calculated for $C_{51}H_{67}N_8O_8PSSi$ 1112.56, found 1112.15.

^{1}H NMR (300 MHz, $CDCl_3$) spectrum of compound **7**:

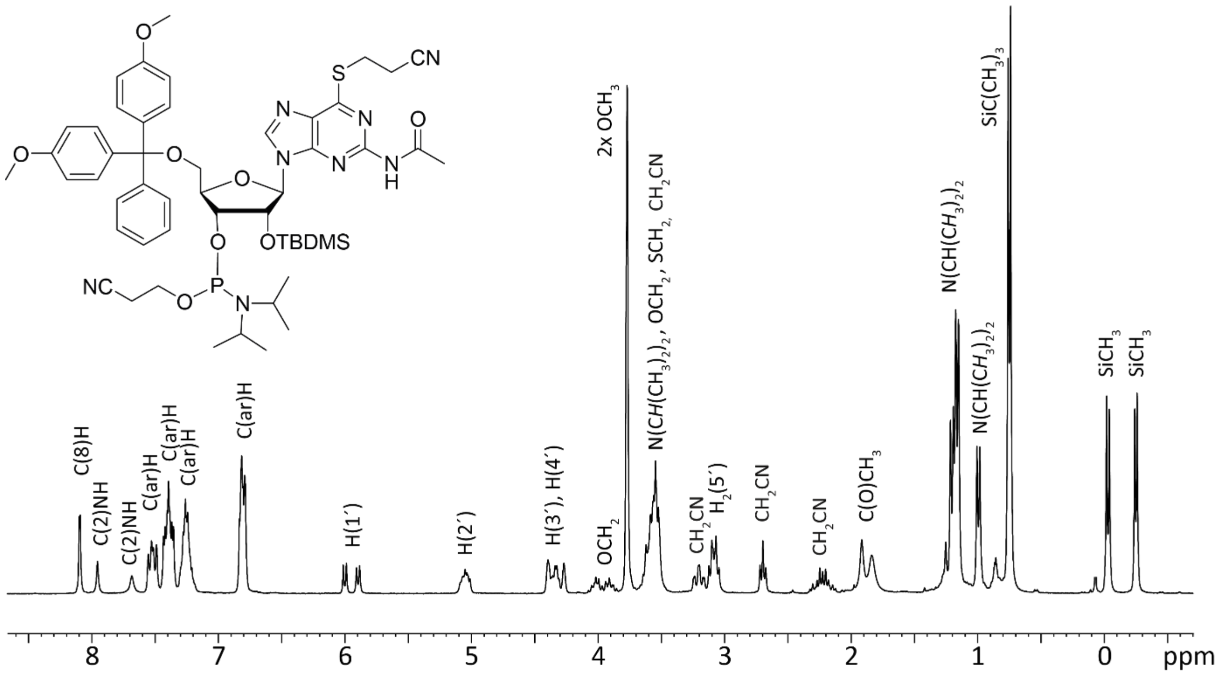

^{31}P NMR (121 MHz, $CDCl_3$) spectrum of compound **7**:

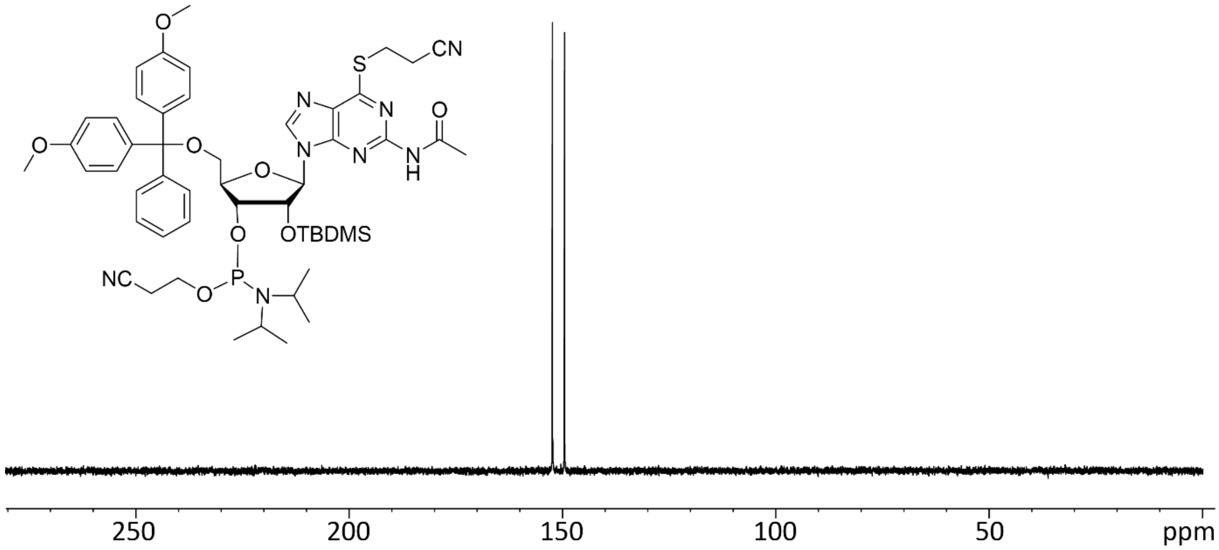

1.3. Preparation of RNA

1.3.1. Solid-phase synthesis of oligoribonucleotides. All oligonucleotides were synthesized on ABI 392 Nucleic Acid Synthesizers, following standard methods: detritylation (130 s) with dichloroacetic acid/1,2-dichloroethane (4/96); coupling (4 x 2.5 s) with phosphoramidites/acetonitrile (0.1 M x 130 µL) and benzylthiotetrazole/acetonitrile (0.3 M x 360 µL); mild capping (2 x 10 s, Cap A/Cap B = 1/1) with Cap A: phenoxyacetic anhydride in tetrahydrofuran (0.2 M) and Cap B: *N*-methylimidazole (0.2 M), sym-collidine (0.2 M) in tetrahydrofuran; oxidation (20 s) with I_2 (20 mM) in tetrahydrofuran (THF)/pyridine/H_2O (35/10/5). The solutions of amidites and tetrazole were dried over activated molecular sieves (3 Å) overnight. Suppliers: 2′-*O*-[(Triisopropylsilyl)oxy]methyl(2′-*O*-TOM)-protected ribonucleoside phosphor-amidites (ChemGenes), standard polystyrene support (*GE Healthcare*, Custom Primer Support^{TM}, PS 200 (40 µmol g^{-1}) or 5G (300 µmol g^{-1}).

1.3.2. Deprotection of 6sG containing oligonucleotides. After RNA assembly, the solid support was treated with a solution of 1,8-diaza-bicyclo(5.4.0)undec-7-ene (DBU) in dry acetonitrile (1 M, 5 mL of a) for 3 hours at room temperature. The solid support was then suspended in a mixture of aqueous NH_4OH (28%, 0.75 mL) and CH_3NH_2 in water (40%, 0.75 mL), additionally containing 1,4-dithiothreitol (50 mM). The reaction mixture was incubated at 65 °C for 1 h. After filtration and evaporation, the crude RNA was dissolved in dry dimethyl sulfoxide (220 µL) and triethylamine trihydrofluoride (TEA*3HF; 275 µL) was added. The reaction mixture was incubated at 3 h at 65 °C overnight. The deprotected RNA was precipitated with sodium acetate (25 µL; 3 M, pH 5.2) and ethanol (750 µL) and the suspension incubated at –20 °C for 2 hours. After centrifugation at 4 °C for 30 min and 11500 rpm, the pellet was suspended in water (1 mL) and stored at –20°C.

Concerning unmodified RNA, the solid support was treated with a mixture of aqueous NH_4OH (28%, 0.75 mL) and CH_3NH_2 in water (40%, 0.75 mL) for 45 min at 65 °C. The supernatant was removed and the solid support was washed 3x with THF/water (1/1, v/v). The supernatant and washings were combined and evaporated to dryness. To remove the 2'-silyl protecting groups, the residue was treated with tetrabutylammonium fluoride trihydrate (TBAF x $3H_2O$) in THF (1 M, 1.5 mL) at 37 °C overnight. The reaction was quenched by the addition of triethylammonium acetate (1 M, pH 7.4, 1.5 mL). The volume of the solution was reduced and applied to size exclusion chromatography (GE Healthcare, HiPrep 26/10 Desalting column; 2.6 x 10 cm; Sephadex G25) by elution with H_2O. The RNA-containing fractions were evaporated to dryness and dissolved in 1 mL H_2O.

Analysis of the crude RNA after deprotection was performed by anion-exchange chromatography on a Dionex DNAPac PA-100 column (4 x 250 mm) at 60 °C. Flow rate: 1 mL min^{-1}, eluent A: 25 mM Tris x HCl (pH 8.0), 6 M urea; eluent B: 25 mM Tris x HCl (pH 8.0), 0.5 M $NaClO_4$, 6 M urea; gradient: 0–60% B in A within 45 min, ultraviolet detection at 260 nm.

1.3.3. Purification of RNA. Crude deprotected RNA was purified on a Dionex DNAPac PA-100 column (9 x 250 mm) at 60 °C with a flow rate of 2 mL min^{-1}. The fractions containing the RNA product were collected and loaded on a C18 SepPak Plus cartridge (Waters/Millipore), washed with 0.1–0.15 M $(Et_3NH)HCO_3^-$ solution and then H_2O. Finally, the purified RNA was eluted with H_2O/CH_3CN (1/1). RNA containing fractions were lyophilized. Analysis of the quality of purified RNA was performed by anion-exchange chromatography as described above; the molecular weights were analyzed by liquid chromatography-electrospray ionization (LC-ESI) mass spectrometry. Yields were determined by ultraviolet photometrical analysis of oligonucleotide solutions.

1.3.4. Mass spectrometry of RNA. All experiments were performed on a Finnigan LCQ Advantage MAX ion trap instrument connected to an Thermo Fisher Ultimate 3000 HPLC system. RNA sequences were analyzed in the negative-ion mode with a potential of –4 kV applied to the spray needle. LC: Sample (200

pmol RNA dissolved in 30 µL of 20 mM EDTA solution; average injection volume: 30 µL); column (Waters XTerraMS, C18 2.5 µm; 1.0 x 50 mm) at 21 °C; flow rate: 30 µL min^{-1}; eluant A: 8.6 mM TEA, 100 mM 1,1,1,3,3,3-hexafluoroisopropanol in H_2O (pH 8.0); eluant B: methanol; gradient: 0–100% B in A within 30 min; ultraviolet detection at 254 nm.

1.4. UV melting analysis

Solutions of duplex or hairpin RNAs were prepared containing 10 mM Na_2HPO_4 and 150 mM NaCl, at pH 7.0 and at RNA concentrations as indicated (usually from 2 µM to 30 µM). Thermal denaturation was monitored on a Cary 100 UV-Vis spectrophotometer equipped with a temperature control accessory. Heating and cooling cycles were performed between 10 to 90 °C three times at a rate of 0.7 °C/min and recorded at 260 nm.

1.5. Crystallography and X-ray analysis

Two synthetic RNA dodecamers, 5'-CGCGAA'UUAGCG-3' (RNA59H) and 5'-CGCGA'AUUAGCG-3' (RNA58H), were used for crystallization trials. RNA was dissolved in water at a concentration of 1 mM, heated at 80 °C for 10 min and cooled at 20 °C at a 1 °C min^{-1} rate. Crystals were grown at 20 °C by the vapor diffusion method using sitting drops by mixing 2 µL of RNA sample with 2 µL of a crystallization buffer made with 10% v/v 2-methyl-2,4-pentanediol (MPD), 40 mM sodium cacodylate pH 7.0, 12 mM spermine, 80 mM sodium chloride, 30 mM magnesium chloride against a reservoir made with 35% MPD. Prior to data collection, crystals were flash-frozen in liquid ethane. X-ray diffraction data were collected on the X10SA beamline at the SLS synchrotron, Villigen, Switzerland. Data were processed with the XDS Package[3] and the structure was solved by molecular replacement with MOLREP[4] using the related PDB 2Q1R RNA model.[5] The structure was refined with the PHENIX package[6] (Supporting Table 1). The model was built using Coot.[7] Coordinates have been deposited with the PDB database (entry numbers D_1292105554 and D_1292105478).

1.6. Primer extension analysis

RNA (10 µL; 1pmol/µL) and 4 µL of an Alexa Fluor 647 5'-end labelled DNA primer (2 pmol/µL; IDT; 5'-/5Alexa647N/CGAATCGTTAGTTCTGTC-3') were annealed for 5 min at 65 °C, then incubated at 35 °C for 5 min and cooled to 4 °C for 1 min (performed in an Eppendorf Mastercycler personal). 8 µL of a mix containing 4 µL of 5 x first strand buffer (250 mM Tris·HCl pH 8.3, 375 mM KCl, 15 mM $MgCl_2$), 1 µL of 0.1 M DTT, 1 µL of 5 mM dNTPs mixture (1.25 mM for each dNTP), 2 µL of DMSO, and 0.4 µL of SuperScript III reverse transcriptase (200 U/µl; Invitrogen) were added to the reactions and incubated at 60 °C for 10 min. Superscript III was used due to its known superior performance in primer extension analyses.[8,9] The primer extension reaction was stopped by addition of 1 µL 4 M NaOH and incubation at 95 °C for 5 min followed by cooling to 4 °C. The Alexa Fluor 647 labelled cDNA strands were precipitated by adding 90 µL of precipitation solution (650 µL water, 150 µL 1 M NaOAc pH 5.2, 10 µL of 20 mg/mL glycogen)) and 250 µL of cold ethanol and stored for 30 min at –20 °C. After centrifugation for 30 min at 4 °C at 13500 rpm, the samples were resuspended in 8 µL of gel loading buffer (97% formamide, 10 mM EDTA). Sequencing ladders were produced by adding 2 µL of 5 mM ddNTPs in addition to the 8 µL of reaction mixture to unmodified RNA samples, prior to incubation at 60°C. Samples were loaded next to a migration control dye (0.1% xylene cyanol, 95% formamide, 10 mM EDTA) on 10% polyacrylamide gels with 7 M urea and run for approximately 100 min at 45 W. The extension products were then analyzed by scanning the gel at 635 nm with a Typhoon FLA 9500 instrument (GE Healthcare).

1.7. PCR-mediated detection of 6sG-to-A' conversion

To determine OsO_4-hydrazine-mediated 6sG-to-A' conversion by PCR-mediated detection, a 47 nt RNA oligo containing a single 6sG at position 16 was synthesized. Furthermore, a synthetic tRNA containing a

single 6sG at position 29 was generated by synthesis of 37 nt and 39 nt half molecules and enzymatic ligation (*S. cerevisiae* $tRNA^{Phe}$ sequence and secondary structure shown in *Supporting Figure 4A*). The OsO_4-treated and untreated RNA was purified, and 100 ng RNA was reverse transcribed using GoScript Reverse Transcriptase (Promega) according to the manufacturer's instructions with a specific stem loop primer complementary to the 3' end of the oligo or the $tRNA^{Phe}$. The generated cDNA was PCR amplified employing primers specific for the universal stemloop sequence and the 3' ends of the tRNA and oligo, respectively (Primers sequences are listed in *Supporting Table 2*). PCR products were subcloned into a pGEM-T-Vector (Promega) and individual clones were picked for colony PCR with M13 primers. PCR products were gel purified according to the manufacturer's instructions (NEB) and subsequently subjected to Sanger sequencing.

1.8. Single and double labeling of HEK293T cells

For single labeling experiments, $1.5x10^7$ HEK293T cells were seeded into 15 cm round cell culture dishes and grown overnight at 37°C and 5% CO_2 in DMEM medium (Gibco). Medium was replaced with DMEM supplemented with 0.025, 0.05 (*Supporting Figure S4*) and 0.1 mM (*Figure 3, Supporting Figure S4*) 6-thioguanosine (6sG; *Figure 3*) or with 0.05 mM 4-thiouridine (4sU; Jena Bioscience; *Supporting Figure S5*). Cells were incubated for 30 min, 1 h or 2 h and subsequently harvested. For pulse-chase experiments (*Supporting Figure S5*), 4sU-containing medium was replaced with DMEM supplemented with 1 mM uridine, and cells were further incubated for 4 h. Samples were collected at 2, 3 and 4 h of uridine chase. For double labeling experiments (*Figure 5*), cells were labeled with 0.1 mM 6sG for 1 h before the medium was removed and replaced by DMEM containing 0.05 mM 4sU. Cells were collected right after 6sG incubation, and at 2 and 4 h of 4sU incubation.

1.9. Proliferation assay

Proliferation of cells treated with different 6sG concentrations (25, 50, and 100 µM) for 30 min or 1 h was monitored for a period of 72 hours by counting cells with a hemocytometer every 24 hours. The mean and standard deviation of four replicates was determined by GraphPad Prism 7 and plotted.

1.10. RNA isolation and RT-qPCR analysis

Total RNA was isolated using the innuPREP RNA Mini Kit (Analytik Jena) according to the manufacturer's instructions. To determine 6sG incorporation efficiency and for amplicon sequencing experiments, mRNA was prepared from total RNA using Magnetic mRNA isolation kit (NEB). For reverse-transcription real time PCR experiments (RT-qPCR), cDNA was generated by GoScript Reverse Transcriptase (Promega) and subjected to real time PCR using Luna® Universal qPCR Master Mix (NEB) with 1.25 ng/ml cDNA and 0.8 mM gene-specific primers in a QuantStudio 3 Real-Time PCR System (Applied Biosystems). Data were normalized against glycerinaldehyde-3-phosphate dehydrogenase (GAPDH) and are expressed as mean ± SEM $2^{-\Delta CT}$ values (n=3). Statistical significance was calculated by unpaired t-test with a significance threshold of $p<0.05$ using GraphPad Prism 7.

1.11. 6sG incorporation analysis by HPLC

Cells were labeled with 0.1 mM 6sG for 2 h before total RNA was isolated and mRNA was prepared as described above. mRNA (2 µg) was digested to mononucleosides as follows: RNA was denatured for 5 min at 95 °C and subsequent cooling on ice. Then RNA was digested with 178 U S1 nuclease (Promega) in a final volume of 50 µl for 4 h at 37 °C, followed by addition of 2 U shrimp alkaline phosphatase (NEB) and 0.2 U phosphodiesterase (Sigma) and further incubation at 37 °C for 1 h. Nucleosides were extracted twice with chloroform and the aqueous phase was analyzed by HPLC on a XBridge C18 5 µm column (4.6 mm x 150 mm) at 35 °C. Elution was in a gradient of 6 column values 0–10% eluent B (acetonitrile) in A (250 mM ammonium acetate, pH 6) at a flow rate of 1 ml min^{-1}. A solution of A, U, C, G, and 6sG ribonucleosides (0.5

mM each; 100 µl) was used as standard. Absorbance was measured at 260 nm and 320 nm (6sG).

1.12. Amplicon sequencing and data analysis

RNA from labeled and unlabeled cells was isolated and treated with OsO_4-hydrazine as described in Materials and Methods. After purification and reverse transcription using GoScript Reverse Transcriptase (Promega) and random hexamer primers, selected targets were amplified with specific primers containing barcode overhangs using standard PCR conditions (primer sequences without barcodes are listed in *Supporting Table 2*). The products were separated on a 1.5% agarose gel, purified from the gel and pooled at equimolar ratio. Library preparation from the amplicon pool and sequencing using the Illumina HighSeq platform with 150 nt paired end reads was performed by Eurofins.

The multiplexed sequencing read data were split into single sample files according to the sample-specific barcodes using flexbar version 3.0.3.[10] The sample-specific sequencing reads were aligned to the respective reference sequences by running bowtie2 version 2.3.4.1[11] in a first round in "end-to-end" mode. Reads that failed to align in "end-to-end" mode were then aligned in a second round by using the "local" mode. Amplicon positions with G-to-A and U-to-C conversions were called using Varscan2 version 2.4.3.[12] We set the maximum depth to 10^6 and the minimum base call quality score to 30. Only U and G positions with a minimum conversion frequency of 10^{-4} were considered further. For identifying the background/baseline mutation/error frequency we analyzed all non U-to-C and non G-to-A changes according to the same criteria as used for U-to-C and G-to-A conversions. In order to minimize errors from potentially misaligned reads we only considered positions on the amplicons which were at maximum 146 bases distant from the amplicon ends. To quantify the number of reads with U-to-C and G-to-A conversions, we used sam2tsv[13] and a custom written perl script (available upon request) to analyze each aligned read and count the U-to-C and G-to-A conversions and the read-specific conversion frequency. Again, only sequence read bases with a minimum base call quality score of 30, and a maximum amplicon end distance of 146 were considered in our analyses.

1.13. Statistical analyses. To determine the statistical significance of differences of G-to-A mutation frequencies of 6sG-labeled and OsO_4-treated versus unlabeled and untreated, and 6sG labeled but not OsO_4-treated samples, and of differences between G-to-A mutation frequencies versus A,C,U-to-N mutation frequencies, Chi-Square analyses with Yates' correction were performed using GraphPad Prism 7. Statistical significance was set to $p<0.05$.

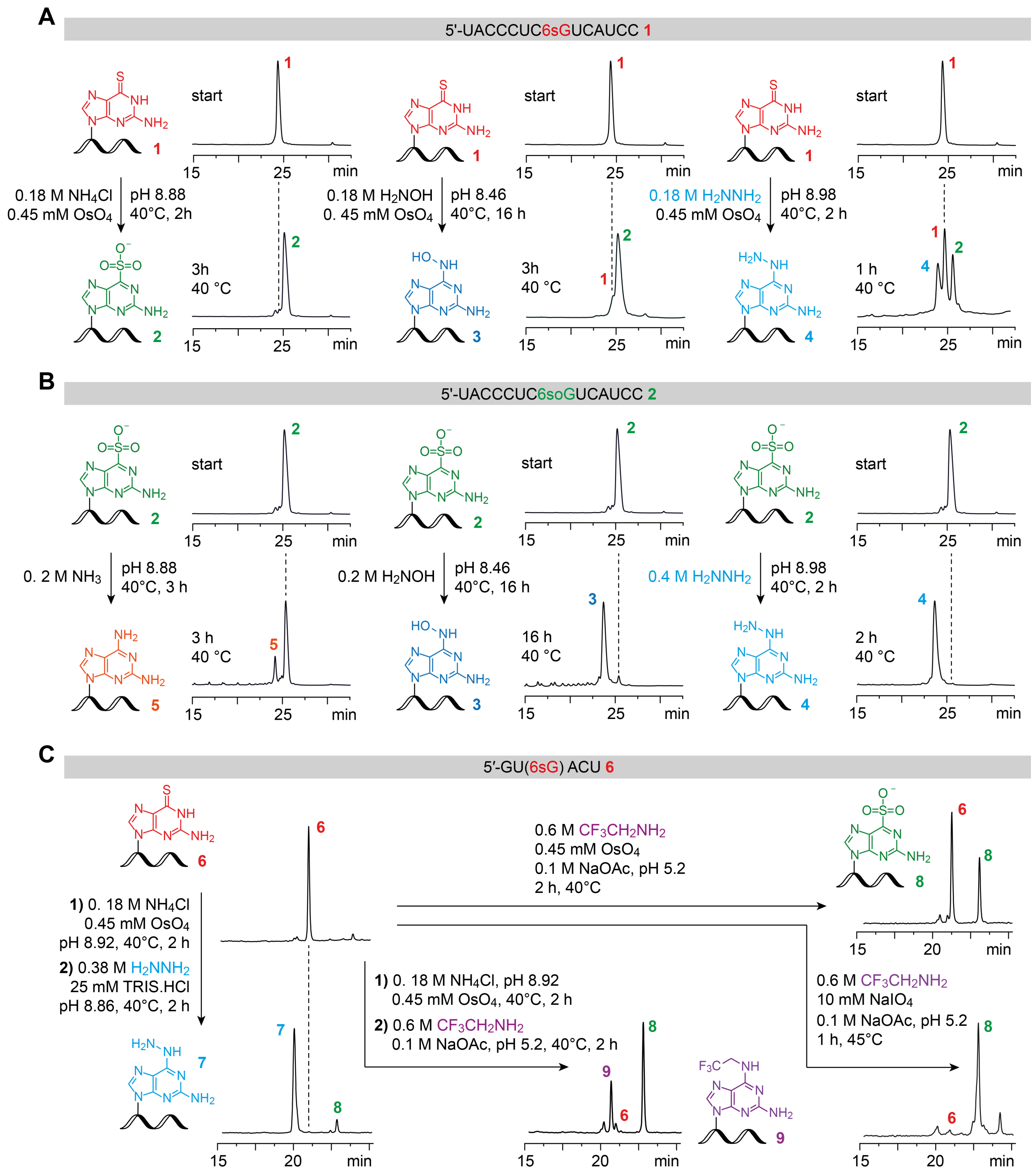

Supporting Figure S1. Optimization of 6sG conversion. (**A**) Attempts for one-step conversion of 6sG into an A analog by simultaneous application of OsO_4 and the nucleophiles NH_4Cl (left), hydroxylamine (middle), and hydrazine (right). Oxidation only or incomplete conversions were observed. Assignments according to mass spectrometric analysis. (**B**) Two-step conversions: After isolation of 6soG RNA (step 1, see panel A, left), different nucleophiles for substitution were tested (step 2). While ammonia resulted in incomplete conversions (left) and degradation during long reaction times (not shown), hydroxylamine substitution required long reaction times (middle); complete conversion was observed for hydrazine in short reaction times. (**C**) Comparison of the optimized OsO_4-hydrazine 6sG-to-A' conversion to OsO_4–$CF_3CH_2NH_2$ and $NaIO_4$–$CF_3CH_2NH_2$ treatments which resulted in incomplete or no conversion.

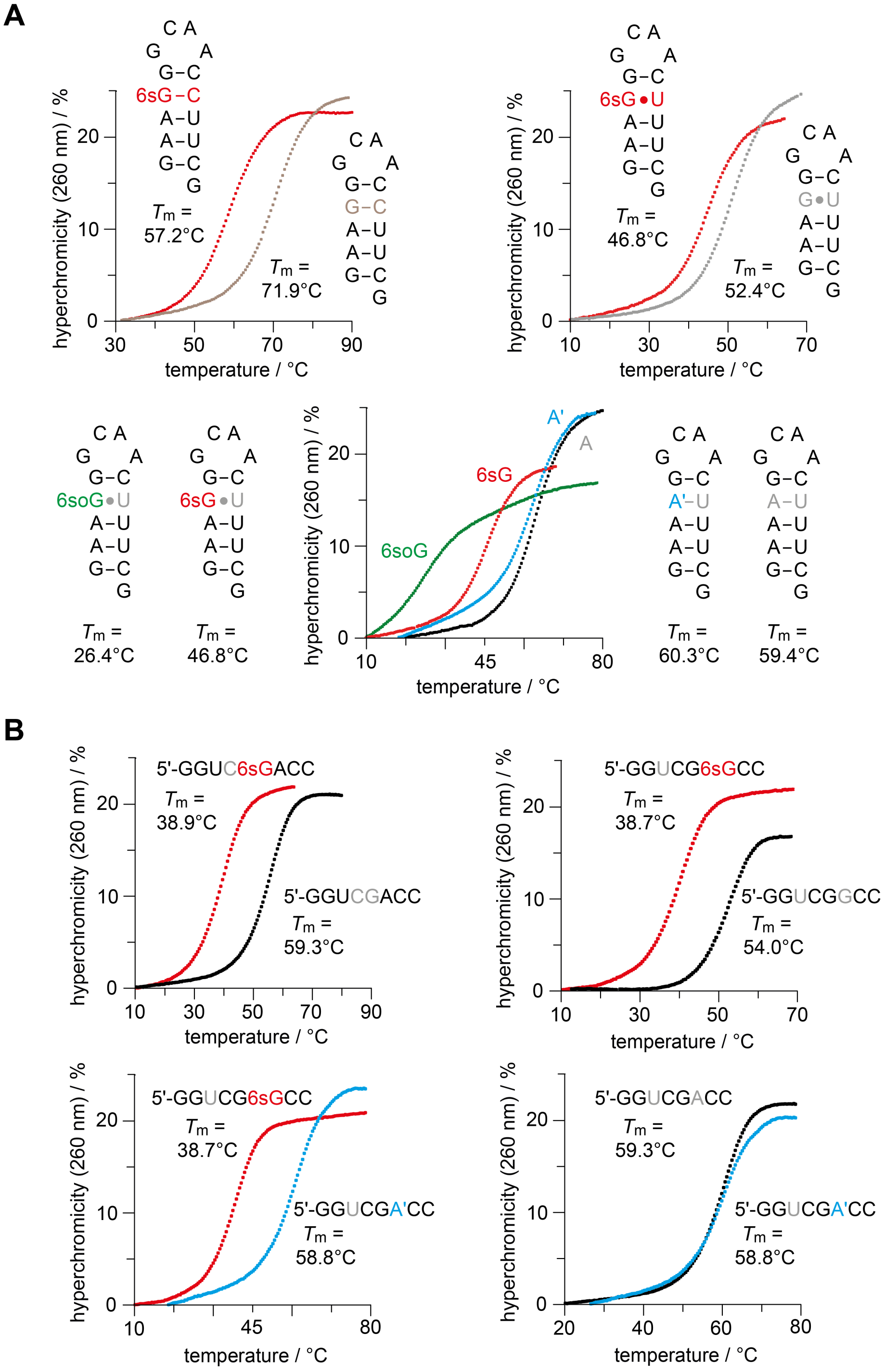

Supporting Figure S2. Melting profiles of 6sG, 6soG, and A'-containing RNA and unmodified counterparts. (**A**) Comparison of base pair constellations within an RNA hairpin. (**B**) Comparison of base pair constellations within a palindromic RNA duplex. The A'-U base pair results in equally stable double helical RNA compared to the native A-U base pair. 6sG and 6soG result in significantly decreased melting temperatures under the following conditions: c(RNA) = 8 µM; 10 mM Na_2HPO_4, 150 mM NaCl, pH 7.0.

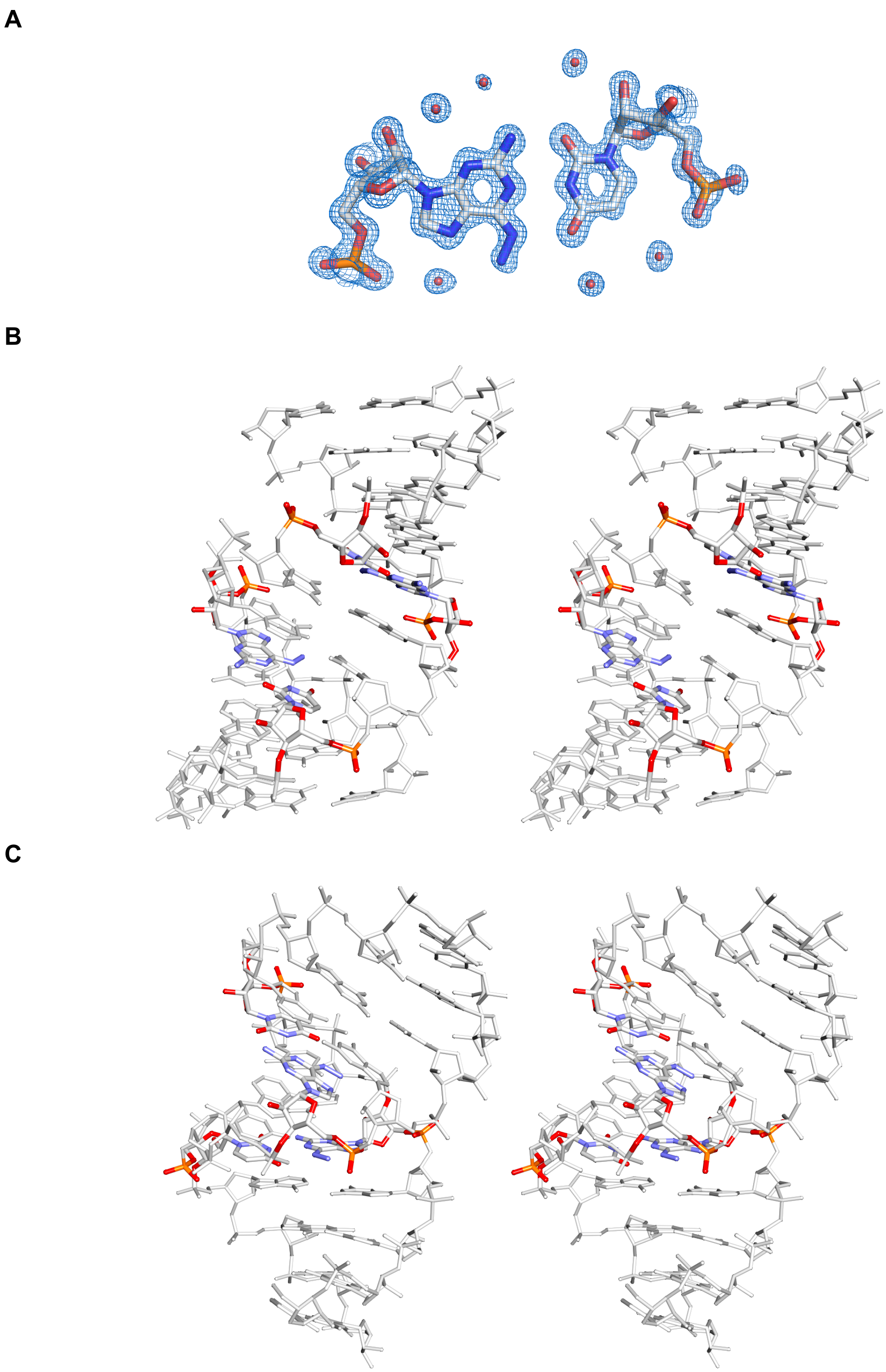

Supporting Figure S3. Crystal structures of A' modified RNAs. (**A**) View of the A'-U base pair observed in the crystal structure of 5'-CGCGAA'UUAGCG (PDB ID 6XUS) at 1.0 Å resolution. The 2Fo-Fc electron density map contoured at the 1.6 σ level is shown in light blue; water molecules are shown as red spheres; (**B**) Two crosses-eyes stereo views of crystal structure of 5'-CGCGA'AUUAGCG (PDB ID 6XUR). The A'-U base pairs (colored) are isosteric to Watson-Crick A-U pairs and integrate well into the double helix; (**C**) Same as (**B**) but rotated by 180 degrees.

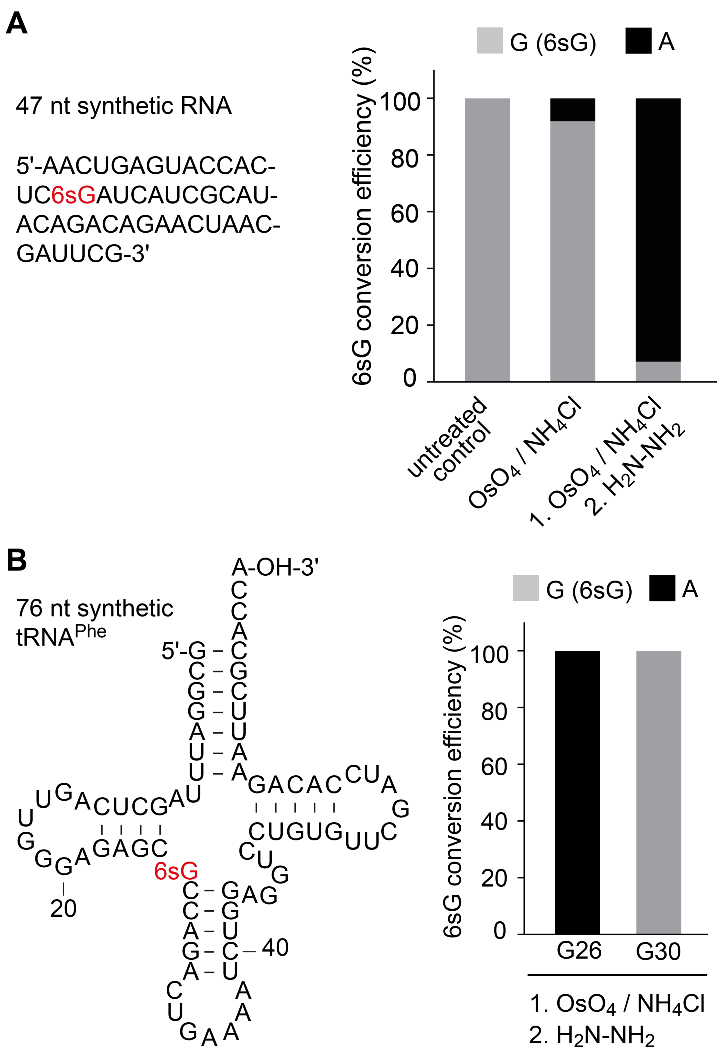

Supporting Figure S4. Conversion of 6sG in structured RNAs. (**A**) Sequence of synthetic 47 nt RNA (left) and conversion efficiency under optimized hydrazine conditions analyzed by Sanger sequencing in comparison to no treatment and OsO_4/NH_4Cl treatment only (right). (**B**) Same as A but for a synthetic full-length tRNA with a single 6sG modification (position 26); conversion under the optimized hydrazine conditions.

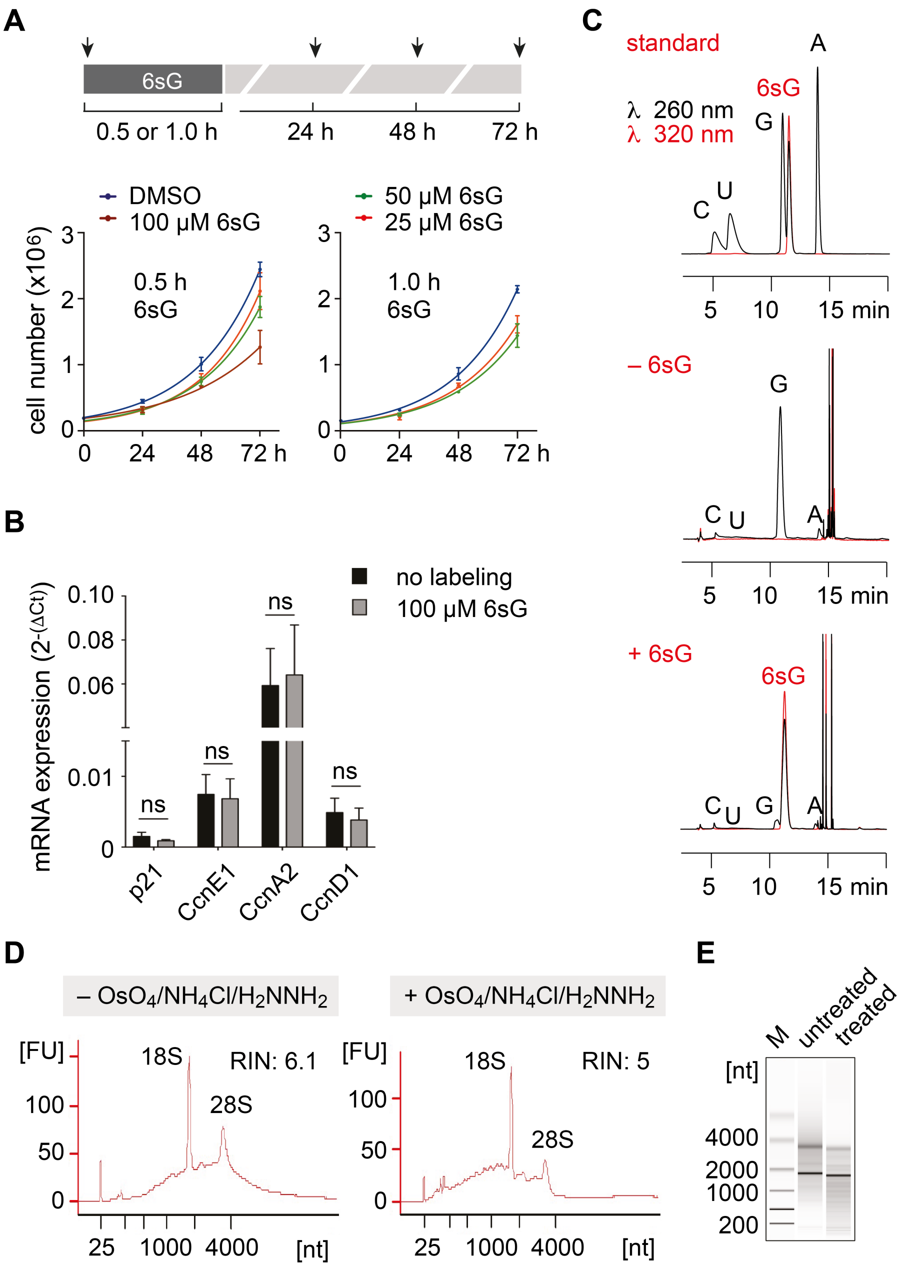

Supporting Figure S5. Characterization of the suitability of 6sG for metabolic labeling. (**A**) 6sG affects proliferation to a minor extent. *Top*, schematic of 6sG labeling conditions and sample collection. Arrows indicate sampling time points. *Bottom*, HEK293T cell numbers at the indicated time points after incubation with different 6sG concentrations for 0.5 h (*left*) or 1 h (*right*). (**B**) RT-qPCR analysis of the indicated transcripts in cDNA from cells incubated with 6sG for 1 h. Mean values ± SEM from three biological replicates are shown. Values were normalized to GAPDH. T-test analysis indicates no significant (ns) differences between 6sG labeled and unlabeled cells ($p>0.05$). (**C**) 6sG is incorporated into cellular mRNA. Mononucleoside preparations from 6sG-labeled (*bottom*) and unlabeled (*middle*) mRNA were separated by RP-HPLC. Defined nucleoside standards were applied as a reference (*top*). 6sG shows maximum absorbance at 320 nm. (**D, E**) OsO_4-hydrazine treatment results in slight degradation of mRNA. Agilent Bioanalyzer profiles (**D**) and virtual electrophoresis pattern (**E***)* of OsO_4-hydrazine treated and untreated enriched mRNA. Peaks of residual 18S and 28S rRNA are shown in (**D**); Note that RIN values reflect the skewed unspecific enrichment of 18S and 28S rRNA by magnetic poly(T) beads; M, molecular size marker.

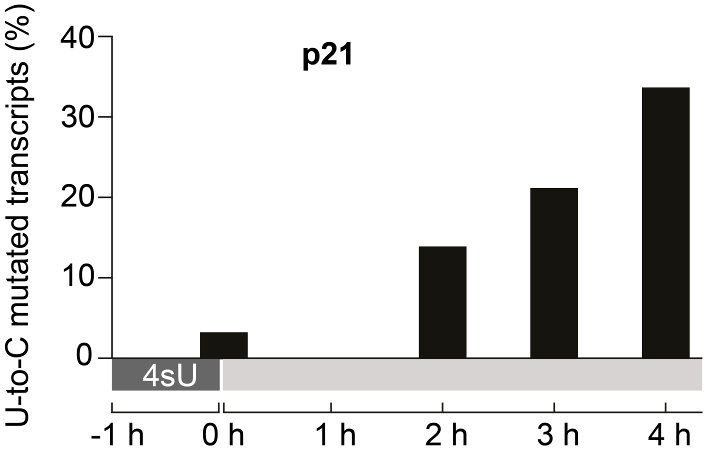

Supporting Figure S6. Ongoing incorporation of 4sU in a pulse-chase labeling experiment. Cells were labeled with 4sU for 1 h, before washout and addition of excess U. OsO_4/NH_4Cl treatment was performed, p21 RNA was analyzed by amplicon sequencing at the indicated time points and the fraction of newly synthesized transcripts was calculated.

Supporting Table 1. X-ray data collection and crystallographic refinement statistics.

		5'-CGCGA'AUUAGCG	5'-CGCGAA'UUAGCG
PDB ID		6XUS	6XUR
Space group		C2	C2
a (Å)		41.42	41.12
b (Å)		35.03	35.14
c (Å)		32.03	31.87
ß		129.72°	129.43°
Beamline		PX II – X10SA	PX II – X10SA
Resolution range (Å)		35.00 – 1.00	35.00 – 1.04
Number of frames		1800	1800
Oscillation angle		0.2°	0.2°
Wavelength Å)		0.9999	0.9999
Average redundancy		6.0	6.3
Completeness [1]		97.1% (76.1%)	99.1% (98.6%)
CC1/2 [1]		99.9 (5.6)	100.0 (14.8)
Average I/σ [1]		9.7 (0.1)	13.32 (0.29)
ISa		23.7	52.2
R/R_{free}		15.4 / 18.3	15.3 / 17.4
Coordinate error (Å)		0.18	0.18
Atoms	RNA	257	257
	Water	77	105
	Ions	2	3
Mean B (Å²)	RNA	15.5	14.3
	Solvent	31.2	31.8

[1] Values for last resolution shell are shown in parenthesis

Supporting Table 2. List of primer sequences. Red letters indicate the universal primer sequence.

tRNA and oligo	
cDNA-stem-loop primer_ tRNA	5‘-CTCAACTGGTGTCGTGGAGTCGGCAATTCAGTTGAGTGGTGGTGCGAAT
cDNA-stem-loop_6sG_Oligo	5’-CTCAACTGGTGTCGTGGAGTCGGCAATTCAGTTGAGTGGCGAATCGTTA
Universal stem loop reverse PCR primer	5’-CACGACACCAGTTGA
6sG_tRNA_fw	5’-GCGGATTTAGCTCAGTTG
6sG_Oligo_fw	5’-AACTGAGTACCACTC
Amplicon Sequencing	
CcnD1_fw	5‘-CGTCCAGGTTCAACCCAC
CcnD1_rev	5‘-GGCCTTATGAACATCCTC
CcnE1_fw	5‘-AAGATGCACACAACATAC
CcnE1_rev	5‘-CACTGGTGTCTGGAGGTG
CcnT1_fw	5‘-CTGGCTTAAGTACCCAAAG
CcnT1_rev	5‘-GCATCCAGAGCTGAGGTG
p21_fw	5‘-CCCAAGCTCTACCTTCCC
p21_rev	5‘-AAGGCAGAAGATGTAGAG
qPCR	
CcnA2_qPCR_fw	5‘-GCAGAGGCCGAAGACGAGAC
CcnA2_ qPCR_rev	5‘-TGAATGGTGAACGCAGGCTGT
CcnD1_ qPCR_fw	5‘-GCCTCACACGCTTCCTCTCC
CcnD1_ qPCR_rev	5‘-GCTTGACTCCAGCAGGGCTT
CcnE1_ qPCR_fw	5‘-GAGTTCTCGGCTCGCTCCAG
CcnE1_ qPCR_rev	5‘-CCTCGCCGTCCTGTCGATTT
p21_ qPCR_fw	5‘-GTACCCTTGTGCCTCGCTCA
p21_ qPCR_rev	5‘-AGATCAGCCGGCGTTTGGAG
GAPDH_ qPCR_fw	5‘-CATCAATGGAAATCCCATCA
GAPDH_ qPCR_rev	5‘-GACTCCACGACGTACTCAGC

4. References

(1) Riml, C.; Amort, T.; Rieder, D.; Gasser, C.; Lusser, A.; Micura, R. *Angew. Chem. Int. Ed*. **2017**, *56*, 13479 – 13483; *Angew. Chem*. **2017**, *129*, 13664 – 13668.

(2) Lusser, A.; Gasser, C.; Trixl, L.; Piatti, P.; Delazer, I.; Rieder, D.; Bashin, J.; Riml, C.; Amort, T.; Micura, R. *Methods Mol. Biol*. **2020**, 2062, 191 – 211.

(3) Kabsch, W. *Acta Crystallogr., Sect. D: Biol. Crystallogr*. **2010**, *66*, 133 – 144.

(4) Vagin, A.; Teplyakov, A. *Acta Crystallogr., Sect. D: Biol. Crystallogr*. **2010**, *66*, 22 – 25.

(5) Li, F.; Pallan, P.S.; Maier, M. A.; Rajeev, K. G.; Mathieu, S. L.; Kreutz, C.; Fan, Y.; Sanghvi, J.; Micura, R.; Rozners, E.; Manoharan, M.; Egli, M. *Nucleic Acids Res.* **2008**, *35*, 6424 – 6438.

(6) Adams, P.D.; Afonine, P.V.; Bunkoćzi, G.; Chen, V.B.; Davis, I. W.; Echols, N.; Headd, J. J.; Hung, L.-W.; Kapral, G. J.; Grosse-Kunstleve, R. W.; et al. *Acta Crystallogr., Sect. D: Biol. Crystallogr*. **2010**, *66*, 213 – 221.

(7) Emsley, P.; Cowtan, K. *Acta Crystallogr., Sect. D: Biol. Crystallogr*. **2004**, *60*, 2126 – 2132.

(8) Lusvarghi, S.; Sztuba-Solinska, J.; Purzycka, K. J.; Rausch, J. W.; Le Grice, S. F. *J. Vis. Exp*. **2013**, *75*, e50243.

(9) Gasser, C.; Gebetsberger, J.; Gebetsberger, M.; Micura, R. *Nucleic Acids Res*. **2018**, *46*, 6983-6995.

(10) Roehr, J. T.; Dieterich, C.; Reinert, K. *Bioinformatics* **2017**, *33*, 2941 – 2942.

(11) Langmead, B.; Salzberg, S. L. *Nat. Methods*, **2012**, *9*, 357 – 359.

(12) Koboldt, D. C.; Zhang, Q.; Larson, D. E.; Shen, D.; McLellan, M. D.; Lin, L.; Miller, C. A.; Mardis, E. R.; Ding, L.; Wilson, R. K. *Genome Research* **2012**, *22*, 568 – 576.

(13) Lindenbaum, Pierre 2015, http://lindenb.github.io/jvarkit/
